# Apigenin: Molecular Mechanisms and Therapeutic Potential against Cancer Spreading

**DOI:** 10.3390/ijms25105569

**Published:** 2024-05-20

**Authors:** Valeria Naponelli, Maria Teresa Rocchetti, Domenica Mangieri

**Affiliations:** 1 Department of Medicine and Surgery, University of Parma, Plesso Biotecnologico Integrato, Via Volturno 39, 43126 Parma, Italy; 2 Department of Clinical and Experimental Medicine, University of Foggia, Via Pinto 1, 71122 Foggia, Italy; mariateresa.rocchetti@unifg.it

**Keywords:** flavonoid, metastasis, chemoprevention, cell signaling pathways, cell growth arrest, programmed cell death

## Abstract

Due to its propensity to metastasize, cancer remains one of the leading causes of death worldwide. Thanks in part to their intrinsic low cytotoxicity, the effects of the flavonoid family in the prevention and treatment of various human cancers, both in vitro and in vivo, have received increasing attention in recent years. It is well documented that Apigenin (4′,5,7-trihydroxyflavone), among other flavonoids, is able to modulate key signaling molecules involved in the initiation of cancer cell proliferation, invasion, and metastasis, including JAK/STAT, PI3K/Akt/mTOR, MAPK/ERK, NF-κB, and Wnt/β-catenin pathways, as well as the oncogenic non-coding RNA network. Based on these premises, the aim of this review is to emphasize some of the key events through which Apigenin suppresses cancer proliferation, focusing specifically on its ability to target key molecular pathways involved in angiogenesis, epithelial-to-mesenchymal transition (EMT), maintenance of cancer stem cells (CSCs), cell cycle arrest, and cancer cell death.

## 1. Introduction

Given its propensity to spread, cancer remains one of the leading causes of death worldwide [1]. Despite numerous advances in cancer treatments, metastatic disease remains the primary cause of death in patients suffering from tumors [2]. Metastatic cascade is a multistep phenomenon in which malignant cells from a primary tumor progressively acquire the ability to infiltrate the surrounding microenvironment, disseminating through the blood and/or lymphatic circuits to reach and colonize distant sites [3]. Therefore, tumor cells must necessarily modify their phenotypic characteristics to adapt to the surrounding microenvironment, proliferate, and evade cell death [4,5,6]. Notwithstanding cytotoxicity and other counterproductive effects, including multidrug resistance, to treat or prevent metastasis, systemic therapeutical approaches such as chemotherapy, targeted therapy, and immunotherapy (often in combination) are currently used [4]. Consequently, it becomes urgent to validate alternative and more effective therapeutic protocols. Dietary phytochemicals, including flavonoids, have the advantage of low toxicity profiles and can reduce multidrug resistance [7,8]. In this regard, given its ability to interfere with various steps of metastatic cascade and correlated cellular signaling pathways, the flavonoid Apigenin seems able to interfere with tumor diffusion [9,10,11]. Thus, in this review, we discuss the role of Apigenin in cancer spreading, addressing specifically its ability to target key molecular pathways involved in angiogenesis, epithelial-to-mesenchymal transition (EMT), maintenance of cancer stem cells (CSCs), cell cycle arrest, and cancer cell death. This is a comprehensive review of selected articles for relevance and impact in oncology research made by searching a combination of keywords such as Apigenin, cancer spread, angiogenesis, epithelial–mesenchymal transition, cancer stem cells, cell cycle, cell death, and apoptosis in the PubMed platform, and including both in vitro and in vivo (animal) studies. In addition, the Clinicaltrials.gov database was searched for clinical trials using the term “Apigenin”. No time restriction was set for the publication date.

## 2. Apigenin

**Figure 1:**
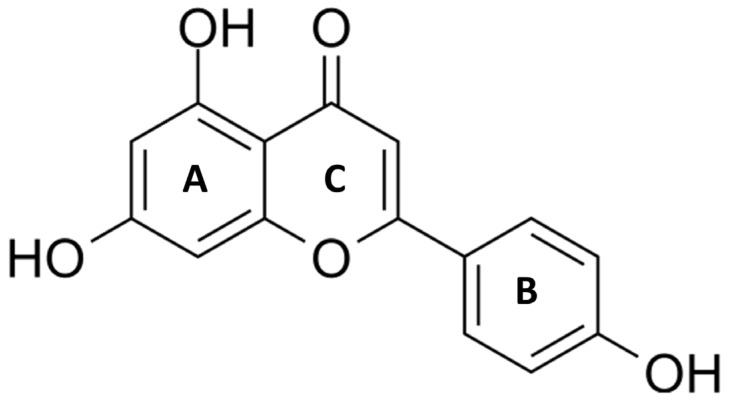
Chemical structure of Apigenin (4′,5,7-trihydroxyflavone). Apigenin is a hydrophobic, naturally occurring flavonoid that consists of two aromatic rings (A and B) linked by three carbons that are in an oxygenated central pyrone ring (C).

Apigenin (4′,5,7-trihydroxyflavone) belongs to a subclass of flavonoids. It is extracted as a yellow crystalline and hydro-insoluble compound [12]. Apigenin has a molecular weight of 270 g/mol and its chemical formula C_15_H_10_O_5_ presents a classic flavone C6-C3-C6 skeleton consisting of two aromatic rings (A and B) linked by three carbons that are in an oxygenated central pyrone ring (C ring), as represented in Figure 1 [12].

Apigenin occurs naturally in a wide variety of edible plants and fruits [13], as detailed in Table 1.

Plant extraction and purification of Apigenin are based on sophisticated biochemical approaches, including high-performance liquid chromatography and solvent extraction methods combined with spectroscopic techniques and absorbance analysis [14,15]. A detailed description of Apigenin’s extraction and purification is beyond the scope of this review. 

Like other flavonoids, Apigenin can be present in foods either in pure form or linked to one or more sugar units [16]. In the glycoside form, Apigenin presents one or more residues of sugar linked to the hydroxyl groups (O-glycosides) or directly to carbon (C-glycosides) [17]. Apigenin (Apigenin-7-O-glucoside), Vitexin (Apigenin-8-C-D-glucopyranoside), isovitexin (Apigenin-6-C-glucoside), rhoifolin (Apigenin-7-O-neohesperidoside), and schaftoside (Apigenin-6-C-glucoside-8-C-arabinoside) represent the more abundant Apigenin glycosides in nature [18]. Data on the pharmacokinetic properties of Apigenin (aglycon and glycoside forms), its bioavailability as well as its metabolism are widely documented [17,19,20]. Despite its hydrophobic nature, many studies are available on the beneficial properties of Apigenin, including its antiviral, antibacterial, antioxidant, and anti-cancer/anti-metastatic effects [12,21,22,23,24]. 

## 3. Apigenin and Angiogenesis

Angiogenesis, the development of new blood vessels from the existing vascular network, is a multistep process precisely regulated by both pro- and anti-angiogenic factors [25]. This phenomenon is an indispensable requirement in several physiological circumstances such as embryonic development, the female menstrual cycle, and pregnancy [25]. In the tumor context, angiogenesis is involved in almost all stages of cancer growth and progression, largely due to chronic oxygen deficiency and the proliferative needs of neoplastic, highly invasive cells [26]. Hence, tumor hypoxia, through the lack of inhibition/degradation of hypoxia-inducible factor 1α (HIF-1α) and its consequent activation, is recognized as the main cause responsible for producing pro-angiogenic factors [27]. As a matter of fact, studies executed by Fu et al. (2022) demonstrated that Apigenin, by favoring HIF-1α demolition and synthesis, inhibited vascular endothelial growth factor A (VEGF-A)/VEGF receptor 2 (VEGR2) and platelet-derived growth factor-BB (PDGF-BB)/PDGF receptor β (PDGFR-β) signaling pathways, with consequent attenuation of angiogenesis both in in vitro and ex vivo assays [28]. The authors further supported these findings by using a pre-clinical human non-small cell lung cancer model in which Apigenin reduced the tumor microvessel density (MVD) and their maturity/stability associated with a reduction in pericyte recruitment coupled with cancer growth suppression [28]. Furthermore, in vitro studies performed by Fang et al. on human ovarian cancer indicated that Apigenin inhibits the transcription of VEGF, in a dose-dependent manner, by interfering with the HIF-1 binding site in the promoters of target genes [29]. More specifically, in this tumor context, Apigenin disturbed the HIF-1α/VEGF axis by perturbing phosphoinositide 3-kinase (PI3K)/protein kinase B (PKB or Akt)/ribosomal protein S6 kinase beta-1 (p70S6K1) and E3 ligase human double minute 2 (HDM2)/p53 signaling pathways [29]. Similarly, Liu et al. showed that Apigenin (in a dose/time-dependent manner) significantly reduced HIF-1α expression and VEGF transcriptional activity in human lung cancer in vitro (A549 cell line) through the inactivation of Akt and p70S6K1 signals; in parallel, the suppression of angiogenesis activity was followed by inhibition of tumor expansion in murine xenografts [30]. Another study demonstrated that a glycosylated form of Apigenin, namely Vitexin, attenuated the protein level of HIF1α in rat pheochromocytoma (PC12) cells under hypoxic conditions by partly altering the Jun N-terminal kinases (JNK) pathway, thus leading to a reduction in transcription of the VEGF gene, followed by reduced cancer invasion *in vitro*. Under the same hypoxic condition, Vitexin decreased the tendency of human umbilical vein endothelial cells (HUVECs) to rearrange into capillary-like structures on a Matrigel layer [31].

**Figure 2:**
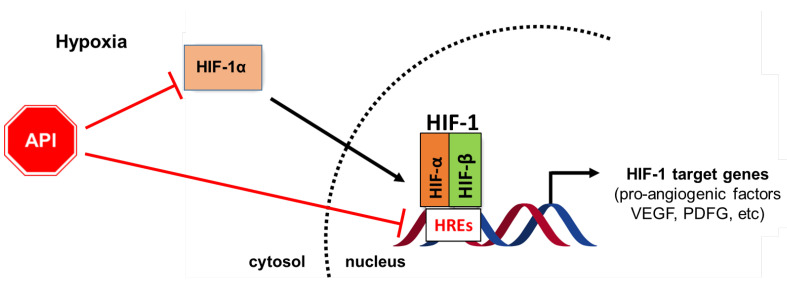
Diagram showing the role of Apigenin (API) in the management of tumor angiogenesis. Apigenin inhibits tumor angiogenesis by targeting HiF-1α/HIF signaling pathways; it also affects the HIF-1/HRE molecular interaction, with consequent transcription suppression of pro-angiogenic target factors, including VEGF and PDGF.

Thus, as explained above, the anti-angiogenesis effects of Apigenin are associated with its inhibitory effects on HIF-1α/HIF activity (Figure 2).

## 4. Apigenin and Epithelial–Mesenchymal Transition

EMT is a process through which epithelial cells adopt the mesenchymal phenotype; it is orchestrated by several cellular events, including the loss of epithelial cell polarity and the disruption of cellular junctions, as well as the rearrangement of the cytoskeletal implicature [32]. Specifically, the EMT is orchestrated by the loss of typical epithelial markers such as E-cadherin, claudins, and zonula occludens-1 (ZO-1), and the enrichment of numerous mesenchymal cell facets, including the expression of N-cadherin and vimentin [33]. Moreover, the EMT process is executed by a plethora of cellular signaling pathways, including transforming growth factor-β (TGF-β), Notch, Wnt/β-catenin, PI3K-Akt, focal adhesion kinase (FAK)/paxillin/ extracellular matrix (ECM), as well as the Hippo-Yes-associated protein (YAP)/ PDZ-binding motif (TAZ) pathways in concert with specific transcription factors such as nuclear factor kappa B (NF-κB), zinc finger E-box binding homeobox 1/2 (ZEB1/2), Snail, SLUG, Twist, and HIF1/2 [33,34]. Physiologically, EMT plays a crucial role during tissue regeneration [35]. In the tumor milieu, this phenomenon orchestrates multiple and interconnected events such as anoikis evasion, acquisition of stemness aspects coupled with high tumorigenicity, and multidrug resistance, as well as adaptation to hypoxia and/or other changes in the tumor microenvironment [36,37]. Therefore, the development of pharmacological strategies that inhibit EMT could have a significant clinical impact on the inhibition of metastasis [38]. Based on these premises, Tong et al. demonstrated the ability of Apigenin to reverse EMT (and its consequences) in human colon cancer both in vitro and in a xenograft model [39]. Increasing doses of Apigenin inhibited cell viability, migration, and invasion of human colon cancer cells (HCT-116 and LOVO), combined with a significant inversion in E-cadherin and vimentin expression; at the same time, the flavone, by inhibiting NF-κB, Snail, Ki-67, NF-κB, and p65 inhibitor of NF-κB kinase (IKK), suppressed tumorigenicity of HCT-116 cells in nude mice xenografts [39]. Similarly, in human hepatocellular carcinoma, Apigenin dose-dependently, by interfering with NF-κB/Snail activity, suppressed cell proliferation, migration, and invasion, and also inhibited tumor growth in vivo [40]. Regarding human prostate cancer, a very interesting study showed that Apigenin was able to suppress EMT either in vitro or in vivo in a dose and time-dependent manner by targeting SPARC/osteonectin, cwcv, and kazal-like domains proteoglycan 1 (SPOCK1)-snail/slug axis [41]. SPOCK1, a glycoprotein isolated from human testes, is a member of the secreted, acidic, cysteine-rich (SPARC) family of extracellular matrix-resident proteins that play multiple roles in cancer progression, including EMT [42,43]. Notably, clinical and pathological studies have demonstrated that SPOCK1 is frequently overexpressed in highly metastatic human prostate cancer tissues, thus it could be an attractive prognostic biomarker and therapeutic target in cancer treatment [44]. In another study, Chen et al. disclosed that the exposure of human cervical cancer (CC) to Apigenin reduces EMT aptitude both in vitro and in a xenograft model [45]. In particular, the authors found the downregulation of FAK (FAK, paxillin, and integrin β1) and PI3K/Akt pathway signaling followed by unbalanced N-cadherin, vimentin, laminin, and E-cadherin levels [45]. Another research demonstrated that both the onco-miRNA miR-152-5p and the Bromodomain-containing protein 4 (BRD4), a member of the bromodomain and extraterminal domain (BET) protein family implicated in histone epigenetic activity, play a role in the metastatic phenotype of CC [46]. Specifically, this condition showed that Apigenin suppressed EMT in CC in vitro and, consequently, blocked cell proliferation and invasion by interrupting the miR-152-5p/BRD4 axis [46]. Furthermore, the treatment of NSCLC—harboring the Epidermal Growth Factor Receptor (EGFR) wild-type phenotype or its mutant—with Apigenin suppressed CD26 expression and the interplay of downstream signaling such as p-AKT and Snail/Slug, resulting in inhibition of EMT-mediated invasion in vitro [47]. In parallel, in an orthotopic mouse model, Apigenin suppressed NSCLC growth and metastasis by targeting the above-mentioned surface antigen [47]. The effects of Apigenin on EMT were evaluated in vitro and in vivo using highly metastatic breast cancer cells (MDA-MB-231 cell line) that endogenously expressed the pro-EMT, interleukin-6 (IL-6) [44]. The results demonstrated that Apigenin, via IL-6 inhibition, decreased the cellular expression of Snail and N-cadherin; on the other hand, the reduced expression of IL-6, phosphorylated Signal Transducer and Activator of Transcription-3 (pSTAT3), phosphorylated extracellular signal-related kinase (pERK), and phosphorylated Akt (pAkt) inhibited tumor growth and invasiveness in xenograft mice [48]. Apigenin exerts an anti-metastatic effect in melanoma due to its interference in STAT3 activation [49]. Specifically, Apigenin not only inhibited lung metastasis of murine melanoma cells (B16F10 cell lines) in vivo but also slowed down human and murine melanoma migration and invasion in vitro [49]. These effects were in part due to decreasing matrix metalloproteinase-2 (MMP2) and matrix metalloproteinase-9 (MMP9) activity, along with unbalanced expression of EMT-associated markers such as keratin-8, E-cadherin, fibronectin, N-cadherin, and Twist [49]. The IκB kinase-α (IKKα) regulates the NF-κB transcription factor that is engaged by epithelial cells during their neoplastic transformation, an event that involves cell survival and apoptosis evasion as well as extracellular matrix lysis, passing across the EMT [33,50]. In this regard, Garcia-Garcia et al. in 2022 demonstrated that Apigenin, by modulating the ectopic expression of IKKα in an in vitro model of skin carcinoma, was able to attenuate the cancer EMT-related malignant facets [50].

Vitexin modulates several functions during tumor diffusion, including angiogenesis, stemness of cancer, and EMT-related events [51]. Thus, the effect of this compound was also tested in human colon cancer cells [52]. In this setting, it was shown that the flavone, by inactivating the PI3K/Akt/mammalian target of the rapamycin (mTOR) signaling pathway, affected EMT-associated features in vitro and in vivo (including the involved markers i.e., E-cadherin, N-cadherin, zonula occludens-1 (ZO-1), Vimentin, Slug, and Snail) and induced apoptosis [52].

**Figure 3:**
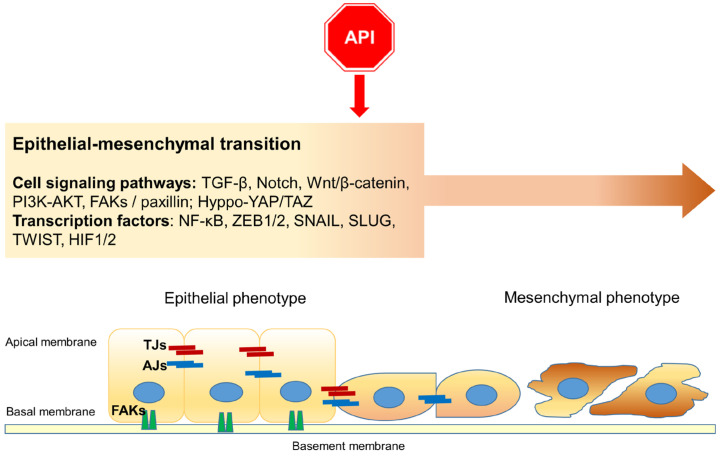
A schematic representation of the main cell signaling pathways as targets of Apigenin (API) during Epithelial–mesenchymal transition (EMT): the flavonoid affects EMT, interfering with specific signaling pathways and transcription factors. Moreover, Apigenin participates in the collapse of cellular junctions. TJs: tight junctions; AJs: adherens junctions; FAKs: Focal adhesions (including integrins and paxilin).

Taken together, this experimental evidence highlights the effect of Apigenin on the EMT phenomenon, providing pivotal details about its antitumor/antimetastatic effects (Figure 3).

## 5. Apigenin Inhibits Cancer Stem Cells

CSCs, also known as “tumor-initiating cells”, represent a small subpopulation of malignant cells with high invasive potential, showing similar facets to normal stem cells, including self-renewal and differentiation aptitudes [53]. Both hypoxia and EMT can contribute to their persistence in tumors [38,54]. Also, CSCs, together with cell death evasion, EMT, and angiogenesis, are recognized as hallmarks of cancer progression, not only because they can contribute to metastases or drive to tumor relapses, but also because they can also contribute to multidrug resistance [55,56,57].

CSCs have been identified in human cancers based on various biomarkers, including cell surface molecules such as cluster of differentiation (CD) 44 and CD133, as well as by several pluripotent transcription factors, such as octamer-binding transcription factor 4 (Oct4), SEX-determining region (SRY) homology box 2 (Sox2), and Nanog, which also regulate their biological activities [58]. Moreover, several aberrant pathways are involved in the maintenance of self-renewal and the differentiation of CSCs, including Wnt/β-catenin, Hedgehog, JAK-signal transducer, and activator of transcription (STAT), Notch, PI3K/Akt/mTOR, NF-κB signaling, and TGF-β, as well as the Hippo-YAP/TAZ pathways [58,59,60,61]. A further distinctive phenotypic behavior of CSCs, showed in vitro, is the propensity to form spheroids (in the case of breast cancer cells, mammospheres); in other words, tumor-derived spheroids are unique because they are purposed for the enrichment of CSCs or cells with stem cell-related characteristics [62,63]. It is reasonable to suppose that differentiating these cells in a quiescent or more mature phenotype can have a crucial therapeutic impact.

Several phytochemicals, including flavonoids, have recently gained considerable attention for their ability to target multiple signaling pathways in CSCs, thereby abrogating their metastatic potential [64,65,66]. In this regard, an in vitro study on human glioblastoma showed that Apigenin, by reducing c-MET expression, downregulated CD133, Nanog, and Sox2 and consequently abolished stem-like features of tumor cells, including their self-renewal ability and invasiveness [67].

As mentioned above, YAP/TAZ is implicated in the self-renewal and tumor-initiation capacities of CSCs [61]. The transcription factors in the TEAD family are the major mediators of YAP/TAZ in terms of transcription and functional outcomes [68]. As a matter of fact, both in vitro and in vivo studies have shown that treatment of highly metastatic human triple-negative breast cancer (TNBC) (MDA-MB-231 and MDA-MB-436 cell lines) with Apigenin interferes with YAP/TAZ-transcriptional enhanced associate domain (TEADs) molecular interactions (as confirmed by monitoring the transcription levels of connective tissue growth factor (CTGF) and cysteine-rich angiogenic inducer 61 (CYR61)—two YAP/TAZ-dependent genes—that in turn inhibited the stemness characteristics of cancer [69]. Additionally, increasing doses of Apigenin inhibited the proliferation, migration, and colony formation of TNBC, hindering mammosphere formation, whereas, at a working concentration of 20 µM, the flavonoid also reduced tumorigenesis in BALB/c nude mice [69]. Similar functional effects of Apigenin were observed by using an additional human breast cancer cell model (MDA-MB-468, TNBC, highly metastatic) whose stemness features were abolished by the inhibition of sirtuin-3 (SIRT3) and sirtuin-6 (SIRT6) protein levels, as supported by in silico analyses [70].

Among other markers such as Nanog, Sox2, and Oct3/4, CD44 is used extensively to verify that isolated subpopulations of normal or cancer cells have stemness features [71]. In this regard, an in vitro study on CD44+ prostate CSCs demonstrated that Apigenin reduced cell migration and arrested the cell cycle, triggering the extrinsic apoptosis pathway; at the molecular level, all these functional effects were attributed to suppression of the PI3K/Akt and NF-κB signaling pathways [72]. Similarly, by suppressing the PI3K/AKT signaling pathway, a glycosylated form of Apigenin (i.e., Vitexin) was able to suppress stemness features of human endometrial cancer, as documented by the downregulation of Oct4 and Nanog [73]. Consequently, different aspects of the malignant phenotype of this cancer were inhibited in vitro by Apigenin, including cell viability as well as proliferative and angiogenetic potential; in parallel was also documented a slowing down of tumorigenesis capability in vivo [73].

The casein kinase 2 (CK2), a multifunctional enzyme involved in cell growth and survival, has become one of the hallmarks of cancer progression [74]. Its activation involves different signaling pathways, such as the Gli1 pathway, contributing to the stemness maintenance of a certain cancer cell subpopulation [74,75]. Indeed, in a paper by Tang et al. (2015)*,* it was shown that Apigenin (in a dose-dependent manner) inhibited the catalytic domain of CK2 (i.e., CK2α) and downregulated GLI Family Zinc Finger 1 (Gli1) expression, affecting the stemness of human ovarian cancer in vitro (SKOV3 cell line) and impeding the self-renewal capacity as well as the propensity to aggregate in spheroids [76]. These data support a previous study in which, similarly, Apigenin, by inhibiting the expression of CK2α, inhibited the proliferation and self-renewal capacity of HeLa spheroids-derived cells [77].

As mentioned before, hypoxic conditions are critical for maintaining CSC features, both in terms of marker expression and self-renewal [54]. In this regard, as shown by Ketkaew et al., Apigenin was able to abolish the hypoxia-induced stem-like phenotype of human head and neck squamous cell carcinoma cells (HN-30 cell line) by reducing the expression of typical stemness markers such as CD44, Nanog, CD105, Oct4, and VEGF [78]. Similarly, Vitexin abolished the stemness of human hepatocellular carcinoma in vitro, as evidenced by the downregulation of the transcriptional activities of ATP-binding cassette subfamily G member 2 (ABCG2), acetaldehyde dehydrogenase 1 (ALDH1), and NANOG genes and by the overexpression of miRNA-34a; this latter event was also responsible for triggering apoptosis, as proven by the increase in Bcl-3 associated X protein (Bax)/B-cell lymphoma-2 (Bcl-2) and Bax/myeloid cell leukemia-1 (Mcl-1) ratios [79]. Accordingly, it is increasingly emerging that dysregulated levels of specific small non-coding RNAs (i.e., miRNA) contribute to regulating some functional aspects of CSCs, including tumor aggressiveness [80].

Interestingly, Apigenin has been shown to enhance tumor susceptibility to common anti-neoplastic drugs, thus eliminating CSCs [81,82]. Indeed, a study showed that Apigenin, by interfering with p53 signaling, was able to attenuate the highly metastatic potential of lung cancer stem cells resistant to cisplatin [81]. Similarly, the addition of Apigenin to cisplatin led to a synergistic effect on prostate cancer stem cells (PCSCs) with cytotoxic and anti-migration activities [82]. Furthermore, in the same context, concomitant treatment with Apigenin triggered apoptosis in PCSCs through downregulation of Bcl-2, upregulation of pro-apoptotic apoptotic protease activating factor-1 (Apaf-1), p21, and p53 expression, and inhibition of PI3K/Akt and NF-κB signaling pathways [82].

The ability of Apigenin to interfere with CSC features is often enhanced by the co-administration of other flavonoids, such as chrysin [83]. For example, a mixture of Apigenin and chrysin showed a synergetic effect in the reduction of colorectal cancer cell clone numbers, as well as the migration and invasion abilities, while increasing cell apoptosis by suppressing the activity of the p38 mitogen-activated protein kinase (MAPK)/Akt pathway [83].

**Figure 4:**
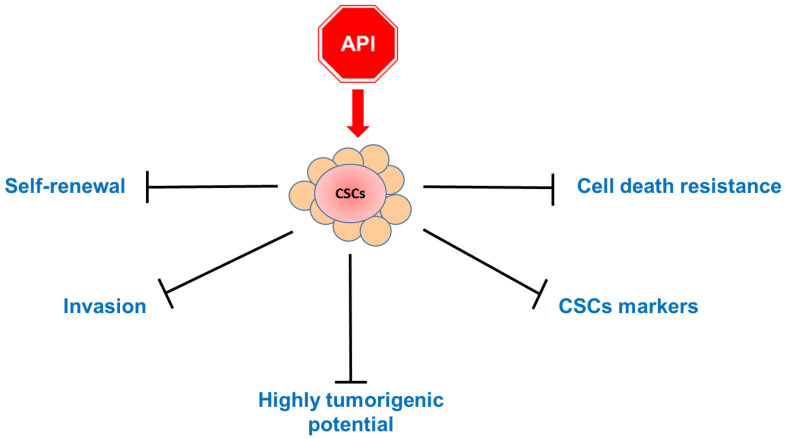
A schematic overview diagram showing the main effects of the treatment of cancer stem cells (CSCs) with Apigenin (API): The apigenin treatment has repercussions on several features of CSC behavior, including propension for self-renewal, invasion, and tumorigenesis. In addition, the flavonoid acts on cell stemness markers, hindering cell death resistance.

To sum up, Apigenin appears capable of attenuating the stemness phenotype of cancer cells by acting on multiple pathways, abrogating their metastatic potential (Figure 4).

## 6. Apigenin and Cell Cycle Arrest

Dysregulation of the cell cycle is one of the main characteristics of cancer that leads to uncontrolled growth and proliferation of abnormal cells that invade and metastasize to different parts of the body. It is known that some of the anticancer effects exerted by flavonoids involve cell cycle arrest and/or triggering cell death [8,84,85,86].

Several investigations have also demonstrated that Apigenin induces anticancer effects in various tumors through cell cycle modulation involving different regulatory pathways [9]. G2/M and G1/S are cell cycle checkpoints that are critical in maintaining DNA integrity and regulating cell growth and proliferation. Indeed, it was demonstrated that Apigenin arrested the human cancer cell cycle at the G2/M phase, the checkpoint that inhibits cells from entering mitosis, which is controlled by cell cycle kinase subunit (Cdc2)/cyclin B as well as by negative regulators such as p21 and p27 [87]. Apigenin induced G2/M phase arrest of the cell cycle in ovarian cancer cells (SKOV-3), as evidenced by flow cytometry [88], and in colon cancer cells (HCT116 line) by increasing the expression of the G2/M cell cycle negative regulators p53 and p21 [89]; in papillary thyroid carcinoma (BCPAP line), Apigenin inhibited growth by decreasing the expression of the G2/M cell cycle positive regulator cdc25C (cell division cycle 25C) [90].

A comparable mechanism was found in pancreatic cancer cells, in which growth was inhibited by Apigenin in a concentration-dependent mode, through the decrease in levels of cyclin A, cell division cycle 25A (cdc25A), cdc25C, and cyclin B [91]. Further studies showed that cancer cell cycle arrest at the G2/M phase through the inhibition of cyclin B-associated cdc2 occurred in many types of cancer cell lines treated with Apigenin, including skin cancer keratinocytes (two murine skin cell lines, C50 and 308) [92], oral cancer cells (squamous carcinoma cell) [93], melanoma cells [94], mouse keratinocytes [95], prostate cancer cells [96], and colon cancer cells [97]. However, in melanoma cells (A375 and C8161 lines), the induction of cell cycle arrest at the G2/M phase was postulated to occur via the Akt/mTOR pathway [98].

In further studies, Tseng et al. showed that Apigenin inhibited breast cancer cell (MDA-MB-231 line) proliferation-inducing G2/M cell cycle arrest through a double action: by suppressing the expression of cyclin A, cyclin B, and cyclin-dependent kinase-1 (CDK1), and upregulating p21, a known downstream effector of the p53 tumor suppressor protein [99]. The same mechanism was found in a study of glioblastoma cells (U87) [100]. In bladder cancer, Apigenin inhibited the proliferation of T24 cells, blocking cell cycle progression at the G2/M checkpoint through an increase in p21 and p27 protein levels and a decrease in Cyclin A, Cyclin B1, Cyclin E, cyclin-dependent kinase-2 (CDK2), Cdc2, and Cdc25C levels [101]. A previous study of T24 cells suggested a different mechanism in the Apigenin-induced increase in the G2/M phase cell population, probably involving a perturbation of the PI3K/Akt pathway [102].

It is known that telomerase activity is a key step in the development of human cancer. It is controlled by the expression of human telomerase reverse transcriptase (hTERT), which is upregulated in many types of cancers, including human malignant neuroblastomas [103,104]. It has been found that Apigenin treatment of human malignant neuroblastoma combined with silencing of hTERT expression (via siRNA technique) reduced the expression of cell cycle regulatory molecules (CDK2, CDK4, and cyclin D1) and dramatically increased the expression of the cell cycle inhibitor p21Waf1, leading to cell cycle arrest at the G1 phase and thereby blocking cell cycle progression from G1 to S phase and inhibiting cancer cell growth [105]. Cell cycle arrest at the G1 phase was also found in human diploid fibroblasts (HDFs) and murine keratinocyte cell line 308 [106]. Apigenin induced G1 arrest through inhibition of cdk2 kinase activity, phosphorylation of retinoblastoma (Rb) protein, and induction of the CDK inhibitor p21Waf1 [106]. The same authors also found that Apigenin can induce skin cancer cell cycle arrest through both mechanisms, inhibiting cell growth at the G1 and G2/M phases [92,106].

An experimental study by Zheng in 2005 demonstrated that Apigenin suppressed the growth of human cervical carcinoma cells (HeLa) by arresting the cell cycle at the G1 phase, with a p53-dependent increment in the expression of p21Waf1 protein [107].

It has been observed that Apigenin can synergistically suppress the growth of cancer cells when combined with other molecules such as drugs. For example, Apigenin combined with a chemotherapy agent, temozolomide (TMZ), was used on glioblastoma cells and showed better performance in cell arrest at the G2 phase compared with Apigenin or TMZ alone, by inhibiting the expression of cyclin D1 [108].

**Figure 5:**
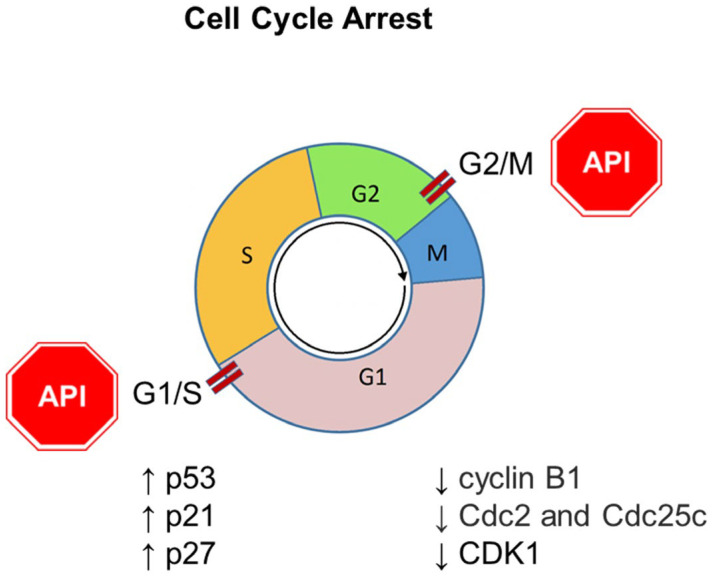
Role of Apigenin (API) in cancer management through the modulation of cell cycle arrest. The downward arrow ↓ represents decreased protein expression; the upward arrow ↑ represents increased protein expression.

In summary, Apigenin counteracts tumor spreading by arresting the cancer cell cycle at both G2/M and G1/S checkpoints (Figure 5).

## 7. Apigenin and Programmed Cell Death

Cell death is known to promote uncontrolled proliferation and is thus one of the main key features of tumor cells [109,110,111,112]. It follows that inducing cancer cell death is currently the primary therapeutic goal of most anti-tumor/metastasis therapies [112,113].

Apigenin has been shown to induce a variety of programmed cell death (PCD) mechanisms, including apoptosis, autophagy, ferroptosis, necroptosis, and anoikis, depending on tumor type as well as the properties of the malignant cells and their microenvironments [110,114,115].

In addition, as explained below, Apigenin can trigger PCD pathways both directly and by potentiating the effects of other anti-cancer drugs [10,12].

### 7.1. Apoptosis

Apoptosis has been identified as the main mechanism of cell death in cancer after treatment with flavonoids, triggered by an intrinsic or extrinsic pathway under different physiological or pathological conditions [8,85,114,115]. When the cell senses intracellular stressors, the intrinsic pathway, also known as the mitochondrial pathway of apoptosis, is activated, leading to permeabilization of the mitochondrial outer membrane and activation of the caspase cascade [115,116]. This process is suppressed by the Bcl-2 protein family, including Bcl-2, B-cell lymphoma–extra-large (Bcl-xL), B-cell lymphoma-w (Bcl-w), and Mcl-1, while Bcl-2-associated death promoter (Bad), Bcl-2 antagonist/killer (Bak), Bax, BH3-interacting domain death agonist (Bid), and Bcl-2 Interacting Mediator of cell death (Bim) cause apoptosis. Activated Bax and Bak mediate the collapse of mitochondrial membrane potential, resulting in the release of cytochrome *c* (Cyt *c*). Apigenin has been shown to induce the apoptosis death pathway by increasing the Bax/Bcl-2 ratio in favor of apoptotic death in several cancer cell lines [45,88,101,117,118,119,120,121,122,123,124,125]. Experimentally, apoptosis has been confirmed via induction of the caspase cascade and DNA fragmentation evident in dying cells [117,126].

One of the most common targets of the antitumor effect of Apigenin is the mTOR/PI3K axis [9,114], which is frequently inhibited by micromolar concentrations of Apigenin in a dose-dependent manner in several cancer cell lines, including bladder, breast, colon, lymphoma, liver, leukemia, prostate, and melanoma [9,98,102,127,128,129]. Furthermore, Apigenin has been reported to influence the PI3K/Akt/mTOR pathway by modulating the expression of key proteins, including phosphatase and tensin homolog (PTEN), Akt, extracellular signal-related kinase (Erk), phosphorylated mTOR (p-mTOR), phosphorylated IKK (p-IKK), and p-p65 [98,130,131,132]. For example, in prostate cancer cells, inhibition of PI3K by Apigenin has been shown to prevent activation of phosphorylation of glycogen synthase kinase-3 beta (GSK-3β), a target of Akt [133,134].

In addition, Apigenin administration increased the expression of forkhead box O3 (FOXO3)—a transcription factor with tumor-suppressing properties that is a downstream target of Akt—via the Akt/PI3K pathway, leading to apoptosis induction in human breast cancer cells [119].

The extrinsic pathway, also known as death receptor-mediated apoptosis, is activated by the recognition and binding of death ligands to cell surface receptors usually belonging to the tumor necrosis factor (TNF) receptor superfamily such as the TNF-related apoptosis-inducing ligand (TRAIL) [116,135,136,137]. This leads to the downstream activation of effector caspases [112,135]. In this regard, Apigenin has been shown to induce cell apoptosis via the extracellular pathway or by both the intrinsic and extracellular pathways [9,11,114,138]. It is important to note that Apigenin has been shown to trigger the extrinsic apoptosis pathway by directly binding and inhibiting adenine nucleotide translocase-2 (ANT2), thereby indirectly enhancing apo2 ligand (Apo2L)/TRAIL-induced apoptosis [139], or by stimulating the upregulation of death receptors 4 and 5 (DR4 and DR5) in a p53-dependent manner, thereby sensitizing NSCLC cells to TRAIL-induced apoptosis [140]. Furthermore, the TRAIL/Apigenin combination was also involved in the upregulation of the Bax/Bcl-2 ratio in a p53-independent manner [141,142,143]. Similarly, Apigenin has been shown to inhibit EGFR and HerB2-mediated phosphorylation of MAPK, Akt, and mTOR signaling pathways, leading to the attenuation of prosurvival protein expression and induction of apoptosis in head and neck and glioblastoma cancer cells [144,145,146]. Additionally, the type I insulin growth factor receptor (IGF-IR) signaling pathway was suppressed by Apigenin both in cell cultures and in prostate cancer xenografts in vivo [133,134]. At the molecular level, Apigenin treatment reduced IGF-IR, Akt, and GSK-3 β phosphorylation, thus suppressing the PI3K-MAPK pathway [133,134]. Furthermore, Apigenin has been shown to induce apoptosis in HeLa cells via apoptosis-stimulating fragment (Fas/APO-1) activation, inducing caspase-3 activation expression and decreasing Bcl-2 levels [107].

The evolutionarily conserved Janus kinase 2 (JAK2)–STAT3 signaling pathway is also used to transduce the binding of external signals to cell surface receptors into the nucleus, modulating a variety of cell responses such as inflammation and cell growth [114,147]; the aberrant regulation of this axis is a hallmark of tumors [11]. STAT3 can be upregulated through the PI3K/Akt pathway [147]. Apigenin can induce apoptosis via inhibition of STAT3 phosphorylation, which greatly influences the chemopreventive effect of this flavone [9,114]. STAT3 has been reported to be a regulator of the expression of membrane metalloproteases (MMPs), Twist1, and VEGF, which are involved in tumor invasion, migration, and angiogenesis [147]. In fact, Apigenin was shown to inhibit VEGF and pSTAT3 expression, leading to the death of several types of cancer cells [148,149,150,151,152,153] and the downregulation of the expression of MMP-2 and MMP-9 [148,149,150,151,152,153,154]. The JAK/STAT pathway plays a crucial role in cytoprotection and inducible nitric oxide synthase (iNOS) and cyclooxygenase-2 (COX2) expression [155]. Apigenin-mediated suppression of JAK/STAT axis has been shown to induce the downregulation of PI3K/Akt in leukemia HL60 cells [156,157] and of COX2, iNOS, and reactive oxygen species (ROS) accumulation in breast cancer cells [158].

The production of ROS is generally increased in cancer cells due to their very high metabolic rates and the hypoxic conditions that support the rapid and massive growth of tumor cells [159]. Depending on the physio-pathological context, flavonoids can act both as pro- and antioxidant messengers [11]. The anticancer activity of Apigenin has been linked to the induction of oxidative stress in cancer cells and the promotion of apoptotic cell death [114]. Furthermore, Apigenin induced apoptosis in human breast, cervical, melanoma, lung, prostate, and head and neck cancer cells [121,138,145,160,161,162,163,164], triggering intracellular ROS accumulation and loss of mitochondrial integrity, as proved by low MMP in Apigenin-treated cells [138,158,163]. Lowering the cell’s antioxidant defense system is another mechanism through which Apigenin increases oxidative stress [9,114]. This has been demonstrated in hepatocellular cancer cells, where catalase and glutathione (GSH), molecules involved in alleviating oxidative stress, were downregulated after Apigenin administration [165]. Consistent with this observation, in breast cancer cell lines and mouse xenografts, Apigenin suppressed the nuclear factor erythroid 2-related factor 2 (Nrf2)-dependent antioxidant system through inhibition of PI3K/Akt axis [166]. Another marker of stress-induced apoptosis is DNA damage induced by ROS overproduction [167], which is often described in Apigenin-treated cancer cells [121,163]. However, in several cancer cell lines, Apigenin-induced DNA damage has been described to be independent of ROS or caspase activity but mediated by p38 and protein kinase C-delta (PKCδ) [168,169,170]. Indeed, the mechanism proposed to explain flavone-induced apoptosis involves the phosphorylation of ataxia-telangiectasia mutated (ATM) kinase and histone H2AX, two key regulators of the DNA damage response, leading to the downregulation of genes involved in cell cycle control and DNA double-strand break repair, rendering cells unable to repair the damage [168].

The tumor suppressor p53 gene, frequently mutated in human cancer cells, is a transcription factor that can modulate the expression levels of several target genes regulating cell metabolism, cell death, and tumor microenvironments [171]. Mutant forms of p53 or wild-type forms are induced by Apigenin [9,114] and accumulate in cancer cells with both antioxidant and pro-oxidant functions [172]. Treating cancer cells with Apigenin was associated with increased mitochondrial apoptosis in several tumors, including breast, bladder, esophagus, mesothelioma, neuroblastoma, prostate, kidney, and thyroid [142,152,161,164,173,174,175,176,177,178]. In addition, mutations that activate the PI3K/Akt pathway and inhibit p53 are mechanisms that are frequently used by cancer cells to evade programmed death [172,179]. Akt has been shown to negatively regulate p53 levels by promoting mouse double minute 2 (MDM2)-mediated targeting of p53 to degradation [172]. Moreover, crosstalk between p53 activation and the STAT3 pathway has been studied recently. For example, in lymphoma cells, Apigenin promoted p53 activation, which mediated ROS reduction through catalase induction and inhibited the prosurvival pathway of STAT3, which has an inhibitory action on p53 [180]. In addition, a study by Kim et al. disclosed that Apigenin induced c-Myc-mediated apoptosis and the phosphorylation of p53 and p38 in anaplastic thyroid cancer cells [181].

NF-κB is a transcription factor that controls many genes involved in proliferation, survival, and inhibition of apoptosis [182,183]. In most cases, Apigenin directly suppresses the activation of the NF-κB signaling cascade in various tumors both in vitro and in vivo [175,184,185] or through the inactivation of IKK [182,183,184,185]. In pancreatic cancer cells, Apigenin caused both a suppression in NF-κB signaling and a decrease in CK2 function, leading to cell apoptosis [186].

Furthermore, inhibition of histone deacetylases (HDACs) is the mechanism through which Apigenin induces apoptosis in prostate cancer cells, both in vitro and in vivo [125,187,188]; however, it should be kept in mind that epigenetic and genetic factors play a fundamental role in tumor initiation and progression [189]. Apigenin has been shown to downregulate telomerase activity by suppressing c-Myc-mediated hTERT expression in leukemia and neuroblastoma cells [190,191].

In addition, Apigenin was shown to inhibit the chymotrypsin activity of the proteasome, allowing a selective increase in the suppressor estrogen receptor-beta (ER-β) and triggering the extrinsic apoptotic pathway in prostate tumors in vitro and in vivo [192]. In breast cancer xenografts, Apigenin has been responsible for proteasome inhibition [193]. An interesting paper showed that Apigenin inhibited interleukin-6 (IL-6) transcription and gene expression in esophageal cancer cells and this mechanism was proposed as a promoter of apoptosis induction [194]. In treated cells, the authors described the induction of cleaved poly (ADP-ribose) polymerase (PARP) and caspase-8 expression, whereas pretreatment of cells with IL-6 completely reversed Apigenin-mediated changes; these data were confirmed with the in vivo antitumor activity of Apigenin in a preclinical nude mouse model [194]. In prostate cancer cell lines (PC3 and LNCaP), the induction of apoptosis by apigenin was associated with increased p21 levels and a significant decrease in polo-like kinase 1 (PLK-1) expression [195]. Co-administration of Apigenin and chemotherapeutic agents has been shown to exacerbate intrinsic apoptosis by increasing oxidative stress and DNA damage [114]. For example, the accumulation of ROS has been described as an apoptotic-inducing mechanism in HeLa cells treated with the combination of Apigenin and paclitaxel by suppressing superoxide dismutase (SOD) activity [196]. Indeed, co-administration with 5-fluorouracil (5-FU) increased the efficacy of Apigenin in human colon cancer through p53 upregulation and ROS accumulation [129,197]. In vivo, combined treatment with Apigenin and 5-FU confirmed significant growth inhibition of hepatocellular carcinoma (HCC) xenograft tumors via activation of the mitochondrial pathway of apoptosis, indicated by activation of caspase 3 and PARP and a decrease in Bcl-2 levels. In particular, this cell death was triggered by increased ROS levels and a decrease in MMP [198]. Furthermore, Apigenin inhibited thymidylate synthase (TS) and forkhead box protein M1 (FOXM1) expression, thereby enhancing the efficacy of 5-FU [197]. The cytotoxicity of 5-FU and cisplatin to the head and neck squamous cell carcinoma cell line SCC25 was enhanced by Apigenin [145]. In addition, a synergistic effect was also described between Apigenin and ABT-263, a BH3 mimetic inhibitor designed to block functions of the pro-survival Bcl-2 family proteins in human colon cancer [199]. Apigenin suppressed the pro-survival regulators Mcl-1, Akt, and Erk, and enhanced ABT-263-induced cell death, resulting in upregulation of Bim and activation of Bax [199]. The PI3K/Akt pathway was shown to be suppressed in HCC doxorubicin (ADM)-resistant BEL-7402 cells via inhibition of the Nrf2 pathway after Apigenin administration [200]. These results were confirmed in vivo since Apigenin and ADM co-treatment inhibited tumor growth and induced apoptosis in BEL-7402 xenografts [200]. A synergistic effect of abivertinib/Apigenin was shown to induce apoptosis and inhibit PI3K/p-Akt/p-IKK/p-p65 activation both in vitro and in vivo in diffuse large B-cell lymphoma xenograft mice [131]. In vitro and in vivo experiments revealed that Apigenin synergistically enhances the cytotoxic effects of Sorafenib, promoting apoptosis in HCC [201,202]. Furthermore, the combination of the small molecule Bcl-2 inhibitor HA14-1 (HA) and Apigenin showed a synergistic effect and caused activation of extrinsic and intrinsic apoptotic pathways compared with treatment alone [203]. Additionally, pretreatment of pancreatic BxPC-3 cells for 24 h with a low concentration of Apigenin and gemcitabine caused the inhibition of the GSK-3β/NF-κB signaling pathway, leading to the induction of apoptosis [120].

Apigenin and Naringenin are two natural compounds with antitumoral properties. In NSCLC cells, compared to monotherapy, co-treatment with Apigenin and naringenin increased the apoptotic rate through ROS accumulation, Bax/Bcl-2 increase, caspase-3 activation, and mitochondrial dysfunction [204].

By modulating molecular pathways similar to those involved in the pro-apoptotic action of Apigenin, data from the literature have also demonstrated the anti-tumor activity of Apigenin derivatives such as Apigetrin (Apigenin-7-O-glucoside) [205,206,207,208] and Vitexin [209,210,211,212,213,214,215,216,217,218].

### 7.2. Autophagy

Autophagy is a catabolic process through which aggregated proteins and damaged organelles accumulated during stress are delivered to lysosomes for digestion [219,220]. Autophagosome formation is a complex mechanism involving several autophagy-related proteins (Atg), including Beclin 1 and light chain 3 (LC3) [221]. A basal level of autophagy can be considered a physiological control mechanism that ensures the maintenance of cellular homeostasis and the growth of all cells. However, in pathological processes, experimental evidence does not always make it clear whether altered autophagy is a protective response to cell damage or, contributes to it [222,223]; due to this ability to drive cells to death, autophagy has been proposed as a cell death mechanism called type II programmed cell death [220,224]. The mTOR complex 1 (mTORC1) signaling pathway is a key sensor of nutrient and energy status and directly phosphorylates the kinase UNC51-like kinase-1 (ULK1), which initiates autophagy in mammals [221]. The PI3K/Akt pathway plays a key role in activating mTOR and inhibiting autophagy. AMP-activated protein kinase (AMPK) is an energy sensor that activates the ULK complex and indirectly promotes autophagy by regulating autophagy-related gene expression downstream of transcription factors, including FOXO3 [223,224].

Several studies have shown that Apigenin-induced autophagy may play a pro-survival role in cancer therapy; in fact, inhibition of autophagy has been shown to exacerbate the toxicity of Apigenin by inducing apoptosis [89,127,225,226]. However, this review will examine studies in which autophagy represents a mechanism of cell death induced by Apigenin.

Apigenin-induced suppression of the Akt/mTOR signaling pathway caused the downregulation of β-catenin in colorectal cancer cells [227] and the increase in ATG5 and LC3-II and the phosphorylation of AMPK and ULK1 in gastric cancer cells [228]. Similarly, in hepatocarcinoma palmitic acid-treated cells, Apigenin restored the blocked autophagic flux by suppressing the PI3K/Akt/mTOR signaling pathway, accumulation of LC3-positive puncta, and lipid degradation [229]. Furthermore, Apigenin inhibited the viability of papillary thyroid carcinoma cells in a dose-dependent manner through the induction of autophagy as shown by Beclin-1 accumulation, LC3 protein conversion, p62 degradation, and significantly increased formation of acidic vesicular organelles. The mechanism induced by flavone involved increased production of ROS followed by DNA damage [90].

In addition, Apigenin-induced autophagy in hepatocellular carcinoma cells was due at least partly to the downregulation of YAP, which is a downstream effector of the Hippo signaling implicated in cancer pathogenesis [230]. Furthermore, Apigenin exerted a pro-autophagic effect mediated by activation of AMPK and direct binding of NRH-quinone oxidoreductase 2 (NQO2) in liver cancer cells [231].

Moreover, Apigenin mediated autophagic cell death via activation of the protein kinase RNA-like endoplasmic reticulum kinase (PERK)-activating transcription factor 4 (ATF4)-C/EBP homologous protein (CHOP) axis, indicating an endoplasmic reticulum stress response, as evidenced by upregulation of glucose regulatory protein 78 (GRP78) and suppression of HIF-1α and enhancer of zeste homolog 2 (EZH2) [228].

A study by Gilardini Montani et al. in 2019 showed the differential effect of Apigenin in two pancreatic cancer cell lines, Panc1 and PaCa44, carrying different p53 mutations (mutp53): Apigenin exerted a stronger cytotoxic effect against Panc1 cells than against PaCa44 cells. Activation of autophagy represents an activated cytotoxic response in Panc1 cells after Apigenin treatment, together with inhibition of mTORC1, reduction of mutp53 and its partial nuclear export, and expression of the chaperone heat shock protein 90 (HSP90). In contrast, mTORC1 activation correlates with the upregulation of HSP90 and the stabilization of mutp53, activating a positive feedback loop between Nrf2 and p62 that triggers the cell antioxidant response [232].

The induction of autophagy has been demonstrated in vitro in human hepatocarcinoma (HepG2 cells) after the administration of Bergamot Polyphenol Fraction (BPF), a mix of six aglyconic flavonoids, including Apigenin, which contributed significantly to the total effect of the mix [233].

Kayacan et al. (2021) showed that co-treatment with Apigenin and curcumin had a synergistic anti-tumor effect in HeLa cells, activating autophagy, apoptosis, and paraptosis and inducing crosstalk between these pathways. In the study, the expression of Atg12, death-associated protein kinase (DAPK), Atg5, Beclin-1, and Bcl-XL was significantly increased [234].

Vitexin has been shown to promote autophagic cell death in colorectal cancer cells and xenograft models [211]. Furthermore, synergistic effects of Vitexin, cinobufacini, and *P. alkekengi* hydroalcoholic extracts were demonstrated in an estrogen receptor (epidermal growth factor receptor 2, EGFR2)-positive breast cancer mouse model [235].

### 7.3. Ferroptosis

Ferroptosis is a non-apoptotic, iron-dependent mechanism of programmed cell death. It is triggered by the accumulation of membrane lipid peroxides to toxic levels arising from oxidative stress, which undermines membrane integrity and triggers osmolytic processes that destroy the cell [236]. This process can be induced by ROS accumulation, GSH depletion, and mitochondrial damage and dysfunction, resulting in reduced or disappeared cristae and mitochondrial size, and changes in mitochondrial membrane fluidity and density characteristic of ferroptotic cells [237,238,239,240]. Experimental studies have shown that induction of ferroptosis can lead to inhibition of tumor cell growth, proliferation, and death, thus playing an important role as an anti-cancer strategy [240,241]. Apigenin has been shown to promote ferroptosis in cancer cells [114].

For example, in multiple myeloma cells, Apigenin suppressed cell growth using different PCD, including apoptosis, ferroptosis, and autophagy [158]. The combination of doxorubicin with Apigenin has shown a synergistic effect in HEK293-STAT1-transfected cells [158]. Similarly, the same group showed that the tumor-suppressing effect of Apigenin-containing chloroform fractions of Thymus vulgaris on multiple myeloma cells can be explained, at least in part, by the activation of ferroptosis [242]. Compared to free Apigenin, Apigenin-loaded magnetic Fe_2_O_3_/Fe_3_O_4_@mSiO_2_ nanocomposites exerted a greater tumor suppressive effect on human lung cancer A549 cells by inducing a ferroptosis death pathway [243]. This process was evidenced by increased levels of ROS and cell lipid peroxidation in A549 cells, as well as an increase in ferroptosis-related proteins, including the inflammation-related protein COX2 and p53, a ferroptosis-mediating gene, and down-regulation of glutathione peroxidase 4 (GPX4) and the anti-inflammatory and anti-apoptotic gene ferritin heavy chain 1 (FTH1) [243].

### 7.4. Necroptosis

Necroptosis is a form of programmed cell death that shows similar facets of necrosis, including inflammation [244,245]. Enlarged cell size and enlarged organelles leading to early membrane rupture are the main features of this PCD. The key players in the necroptotic machinery are receptor-interacting serine/threonine kinase 3 (RIPK3) and mixed lineage kinase (MLKL). MLKL is phosphorylated by RIPK3, then oligomerized and translocated to the plasma membrane where it interacts with certain membrane phospholipids, increasing their permeability and allowing the release of pro-inflammatory cytokines and chemokines [244]. The necroptotic pathway can be triggered by different stimuli, including members of the tumor necrosis factor receptor (TNFR) superfamily, pattern recognition receptors (PRRs), T cell receptors (TCRs), and chemotherapeutic drugs [114,244,245]. As necroptosis is often described in cells lacking functional death receptors of the apoptotic pathway, it serves as an alternative or safety mechanism to promote cell death; in this sense, it can halt tumor development and can be considered an anticancer mechanism [234,235]. On the other hand, when considered as a form of necrotic death, it can induce an inflammatory response that promotes cancer progression and metastasis [246,247].

Apigenin has been shown to induce ROS accumulation, mitochondrial dysfunction, and ATP depletion, leading to apoptosis and necroptosis, as evidenced by increased levels of cleaved caspase-3 and its substrate PARP, and the Bax/Bcl-2 ratio in human mesothelioma cells [248]. In these cells, Apigenin also increased the expression of the necroptosis mediators p-MLKL and p-RIP3 while normal mesothelioma cells were not affected by the flavone [248]. The combination of metformin and Apigenin caused ROS-induced DNA damage and promoted apoptosis, autophagy, and necroptosis in human pancreatic cancer cells [249]. Similar results were obtained from a xenograft cancer model, where the combination of metformin and Apigenin synergistically reduced tumor size and weight [249].

Apigetrin, a stable natural flavonoid with better solubility compared to Apigenin [205], showed antitumor activity and induced necroptosis in human HCC cells [206].

### 7.5. Anoikis

Anoikis is a form of PCD used to eliminate detached or misplaced cells under physiological or pathological conditions [114]. It occurs when cells lose interactions with the normal extracellular matrix (ECM) and fail to receive the biochemical and mechanical signals they need to survive and grow; thus, anoikis prevents cells from growing and implanting in inappropriate places, such as other organs, where they could cause damage [250,251]. During the metastasis process, tumor cells need to be able to overcome anoikis and survive in an ECM-depleted environment [252]. Similar to the apoptotic cascade of endonuclease activation, DNA damage, and cell death, the initiation of anoikis is facilitated by intrinsic and extrinsic caspase activation [253]. The proteins of the Bcl-2 family are key players in both these processes [253].

In human cutaneous melanoma cells, Apigenin caused cell proliferation inhibition and anoikis induction without affecting normal cells [254]. In particular, the main effects of Apigenin administration were the reduction in integrin protein levels and the inhibition of the phosphorylation of FAK and Erk1/2, inducing anoikis. Increases in caspase-3 and cleaved PARP were associated with the induction of anoikis [254]. Similar results were described by Hu et al. who showed that Apigenin inhibited the expression of FAK in ovarian cancer cells, resulting in the inhibition of in vitro migration and invasion and in vivo metastasis [255]. A similar effect of cell migration inhibition, consistent with an anoikis mechanism, has been described in Apigenin-treated cervical cancer cells, where downregulation of FAK and PI3K/Akt signaling led to cancer cell death [45].

Apigenin has been shown to inhibit hepatocyte growth factor (HGF)-induced invasive growth of human breast cancer cells, including motility, spreading, migration, and invasion. Apigenin suppressed HGF-induced activation of the PI3K/Akt pathway as well as integrin β4 function, thereby reducing lung colonization of metastatic tumor cells in nude mice and spontaneous intravasation and organ metastasis in chick embryos [256].

In summary, Apigenin can modulate several signaling pathways, leading to different types of cell death that are highly dependent on the type of tumor cell (Table 2). However, different forms of cell death interact in tumors and can have both synergistic and opposing effects on cancer cell survival. The molecular mechanisms of these pathways often overlap, resulting in crosstalk that is difficult to understand and requires an integrative approach and analysis to fully unravel.

## 8. Conclusions and Perspectives

The currently available synthetic preventive and anti-metastatic chemotherapeutics are often expensive, although they are very effective and present non-negligible toxic effects. In this scenario, Apigenin fits favorably as a chemotherapeutic agent affecting tumor cell survival, both directly and indirectly, inhibiting invasion and metastasis without significant toxic effects on normal cells. As explained in this review of both in vitro and in vivo studies, by modulating different signal pathways, Apigenin can induce cell cycle arrest, trigger programmed cell death, stop tumor-associated angiogenesis, and affect both EMT and invasive potential of CSCs. Furthermore, as an adjuvant, Apigenin enhances the therapeutic efficacy of conventional anticancer drugs [81,82,108,120,129,131,145,158,196,197,200,201,202,203,243]. Of note, Apigenin, like other dietary flavonoids, has the advantage of being inexpensive and readily available and has a low toxicity profile [8,259]. However, despite the multiple anti-cancer therapeutic properties of this compound, its biological applications are limited by its hydrophobic nature and consequently, its bioavailability. This last aspect limits its clinical use; thus, to improve the bioavailability of this compound, several alternatives are being developed for new formulations, including nanoparticles and similar devices [260]. Although Apigenin is recognized as a promising pharmaceutical agent, clinical studies on its anti-cancer effects are very limited. Only one clinical trial (NCT00609310, clinical trials.gov), on the effects of Apigenin in association with another flavonoid (epigallocatechin gallate) on the recurrence rate of colorectal carcinoma, has been reported. In addition, contrasting results from two association studies revealed that dietary intake of certain flavonoids, including Apigenin, may reduce ovarian cancer risk in a prospective study of almost 70,000 women [261], while not supporting the risk of cancers (breast, colorectal, lung, endometrial, ovarian) in a prospective study of about half of the cases (40,000 women) [262]. This implies that additional long-term prospective studies and clinical trials are necessary before introducing this valuable flavone into clinical practice for cancer management.

## Figures and Tables

**Table 1:** Food sources containing the highest amounts of Apigenin.

Source	Concentration (µg/g)
Dried parsley	45,035
Dried chamomille flower	3000–5000
Parsley	2154.6
Celery seed	786.5
Vinespinach	622
Chinese celery	240.2
Kumquats	218.7
Celery	191
Dried oregano	177.1
Artichoke	74.8
Juniper berries	72.6
Peppermint	53.9

**Table 2:** Cell death induced by Apigenin in different types of cancers.

	Types of Cancer	Cells/Animal Model	Molecular Pathway/Protein	Ref.
Apoptosis	bladder	T-24	↑Bax/Bcl-2; ↑Bad; ↑Bak; ↑caspase-9, ↑caspase-3; ↑caspase-7; ↑c-PARP	[101,102]
		RT112	↑c-PARP; ↑fragmented DNA	[117]
	breast	SK-BR-3	↑p53; ↑Bax; ↑Cyt *c*; ↑LDH	[152]
		BT-474, MDA-MB-453, MCF-7, T47-D, HBL-100	↑Cyt *c*; ↑caspase-3; ↑fragmentated DNA	[126]
		T47D, MDA-MB-231	↓PI3K/Akt/mTOR; ↑Bax/Bcl2	[127]
		MDA-MB-231/xenograft	↓proteasome; ↑Bax; ↑c-PARP	[193]
		MCF-7, Hs578T, MDA-MB-231	↓PI3K/Akt; ↑FOXO3a;	[119]
		MCF-7	↑ROS	[160]
		4T1/xenografts	↓PI3K/Akt/Nrf2	[166]
		MCF-7	↑ROS; ↑p53; ↑Bax/Bcl-2; ↑c-PARP; ↑caspases	[161]
		SKBR3; BT-474; MDA-MB-453	↓JAK2/STAT3/VEGF; ↑c-PARP; ↑caspase-8; ↑caspase-3	[148,149,150,176]
		MDA-MB-453	↓HER2; ↓JAK1; ↓STAT3; ↑p53	[150,176]
	cervical	HeLa, SiHa, CaSki, C33A	↑ROS	[162]
		HeLa	↑Fas/APO-1; ↑caspase-3; ↓Bcl-2	[107]
		HeLa, C33A	↑Bax/Bcl-2	[45]
	colon	HCT116	↑caspase-8; ↑caspase-9; ↑caspase-3; ↑c-PARP	[89]
		HT-29/xenografts	↓mTOR/PI3K/Akt; ↑Bax/Bcl-2; ↓CCND1	[128,129]
		HT29, DLD-1, COLO320 and HCT116	↓Mcl-1; ↓Bcl-xL; ↓STAT3	[153]
		HCT-116, SW480, HT-29, LoVo/xenografts	↑NAG-1; ↑p53; ↑PKCδ; ↑ATM	[169]
	esophageal	OE33, KYSE-510	↑caspase-9; ↑caspase-3; ↑p53	[173,174]
		Eca-109, Kyse-30	↓IL-6; ↑c-PARP; ↑caspase-8	[194]
	gastric	HGC-27, SGC-7901	↑Bax/Bcl-2; ↓MMP; ↑caspase-3	[124]
	head and neck	CAL-27, Scc-15 and FaDu	↓Akt; ↓Erk1/2; ↓EGFR; ↓ErbB2	[144]
		SCC25	↑TNF-R; ↑TRAIL-R; ↓Bcl-2; ↑caspase-3 ↑ROS; ↓GSH	[145]
	leukemia	K562	↑c-PARP; ↑ DNA fragmentation	[117]
		HL60	↓JAK/STAT; ↓PI3K/PKB; ↑caspase-8; ↑caspase-7	[156,157]
		THP-1	↑DNA damage; ↑ATM; ↑H2AX; ↑p38; ↑PKCδ	[168]
		THP-1, U937, HL60, Jurkat, K562	↑caspase-3; ↑PKCδ	[170]
		U937, THP-1 and HL60	↓telomerase; ↑caspase	[191]
	liver	HepG2	↑ROS; ↓catalase; ↓GSH	[165]
		HepG2	↓PI3K/Akt/mTOR; ↑caspase-3; ↑caspase-9; ↑c-PARP; ↑Bax/Bcl-2	[226]
		BEL-7402/xenografts	↓PI3K/Akt/Nrf2	[200]
	lung	H460, A549	↑c-PARP; ↑Bax/Bcl-2; ↑caspase 3	[121,122,123]
		A549	↑ROS; ↑DNA fragmentation; ↑Cyt *c*; ↑AIF; ↑Endo G ↑Bax/Bcl-2, ↑caspase 3, ↑caspase-9, ↑PARP	[163]
		A549, H1299	↑Bad; ↑Bax/Bcl-2, ↑Bcl-xL; ↑DR4; ↑DR5; ↑TRAIL	[140]
		NCI-H23	↑PTEN; ↑Bax/Bcl-2; ↑caspase 3	[130]
	lymphoma	U2932, OCI-LY10	↓PI3K/Akt/mTOR; ↓p-mTOR, ↓p-AKT, p-↓IKK, ↓p-p65	[131]
		BC3, BCBL-1	↓Akt/mTOR; ↓ROS; ↑p53; ↑catalase; ↑c-PARP	[183]
	melanoma	A375	↑ ROS; ↑Cyt *c*; ↑Bax/Bcl-2, ↑caspase 3, ↑caspase 9, ↑PARP; ↓MMP; ↑DNA fragmentation	[163]
		A375, C8161	↑caspase-3 ↑c-PARP ↓(p)-ERK1/2, ↓Akt; ↓mTOR	[132]
	mesothelioma	MM-F1, MM-B1, H-Meso-1/xenografts	↑Bax/Bcl-2; ↑p53, ↑caspase 8; ↑caspase-9, ↑c-PARP; ↓AKT, ↓c-Jun; ↓NF-κB	[177]
	multiple myeloma	U266, RPMI 8226	↓Mcl-1, ↓Bcl-2, ↓Bcl-xL, ↓XIAP, ↓Survivin; ↓CK2; ↓Hsp90/Cdc37/Cdk4	[257]
		NCI-H929	↓STAT1/COX-2/iNOS; ↑ROS; ↓MMP	[244]
	neuroblastoma	NUB-7, LAN-5/xenografts	↑p53; ↑Bax; ↑caspase-3	[180]
	ovarian	A2780, OVCAR-3, SKOV-3	↑ROS; ↓ROS; ↑caspase	[258]
		SKOV	↑caspase-9; ↑caspase-3; ↑Bcl-2	[85]
	prostate	22Rv1/xenografts	↑p53; ↑ROS; ↑Cyt *c*; ↑caspase-3, ↑Bax/Bcl-2	[164]
		PWR-1E, LNCaP, PC-3, DU145	↑ROS; ↑caspase 3; ↑caspase-7; ↑caspase-8; ↑caspase-9; ↓c-IAP2; ↓MMP: ↑Cyt *c*	[140]
		LNCaP	↑Bax/Bcl-2	[119]
		PC-3, DU145	↓XIAP; ↓c-IAP1; ↓c-IAP2; survivin; ↑Bax/Bcl-2; ↑Cyt *c*; ↓HDAC1	[125]
		PC-3, 22Rv1/xenografts	↓HDAC1; ↓HDAC3; ↑Bax/Bcl-2	[187]
		LNCaP, PC-3	↑p21; ↓PLK-1	[195]
		DU-145, PC-3/xenografts	↓proteasome; ↑ER-β; ↑caspase-8; ↑caspase-3	[192]
		DU-145, PC-3	↓IFG-IR/PI3K/MAPK; ↓IGF-IR; ↓Akt; ↓GSK-3β; ↓cyclin D1; ↑p27/kip1	[133,134]
		PC3	↓Akt; Bad	[132]
		PC-3, 22Rv1/TRAMP mice	↓NF-ĸB; ↓IKKβ	[184,185]
		DU145	↓ANT2	[139]
	pancreas	MiaPaCa2, DanG	↓NF-ĸB; ↓CK2; ↓p-Bid; ↑caspase-8	[186]
		BxPC-3, PANC-1	↑Cyt *c*; ↑caspase 9; ↑caspase 3	[120]
	renal	ACHN/xenografts	↑p53; ↑Bax; ↑caspase-9; ↑caspase-3	[178]
	thyroid	FRO	↑c-Myc; ↑p38; ↑p53	[181]
Autophagy	colon	HCT-116, SW480	↓β-catenin; ↓Akt/mTOR	[227]
	gastric	AGS, SNU-638	↑PERK-ATF4-CHOP; ↑GRP78; ↓HIF-1α ↓Ezh2; ↑Atg5; ↑LC3; ↑AMPK and ↑ULK1; ↓p-mTOR; ↓p62	[228]
	liver	SMMC-7721, SK-HEP1	↑LC3-II; ↑ULK1; ↓SQSTM1/p62; ↓YAP	[230]
		HepG2	↑LC3-II/LC3-I; ↑AMPK; ↑NQO2	[231]
	pancreas	AsPC-1	↑AIF; ↑p62; ↑LC3-II	[232]
	thyroid	BCPAP	↑LC3-II; ↑beclin-1; AVO accumulation	[90]
Ferroptosis	myeloma	NCI-H929	↓MMP; ↑LC3-II; ↑beclin-1; ↑ROS	[242]
		HEK293	↓MMP; ↑caspase-3; ↑caspase-9; p38; ↑LC3-II; ↑beclin-1; ↑ROS; ↓Akt; ↓COX-2; ↓iNOS	[158]
	lung	A549	↑ROS; ↓GPX4; ↓SOD; ↑Bax; ↑caspase-3; ↑caspase-8	[243]
Necroptosis	mesothelioma	MSTO-211H, H-2452	↑p-MLKL; ↑p-RIP3	[248]
	pancreas	AsPC-1	↑MLKL; ↑p-MLKL; ↑RIP3; ↑p-RIP3	[249]
Anoikis	breast	MDA-MB-231/xenografts	↓PI3K/Akt; ↓integrin β4	[256]
	cervical	C33A	↓PI3K/Akt; ↓FAK	[45]
	melanoma	A2058, A375	↑caspase-3; ↑c-PARP; ↓FAK; ↓ERK1/2; ↓Integrin	[254]
	ovarian	A2780/xenografts	↓FAK	[255]

AIF, apoptosis-inducing factor; AMPK, 5′ adenosine monophosphate-activated protein kinase; ANT2, Adenine nucleotide translocase 2 Atg5, autophagy-related 5; ATF4, activating transcription factor 4; ATM, ataxia telangiectasia mutated; Akt/PKB, protein kinase B; APO-1, apoptosis antigen 1; Avo, Acidic vesicular organelle; Bad, Bcl-2-associated death promoter; Bak, Bcl-2 antagonist/killer; Bax, Bcl-2 associated X protein; Bcl-2, B-cell lymphoma-2; Bcl-xL, B-cell lymphoma extra-large; Bid, BH3-interacting-domain death agonist; CCND1, Cyclin D1; Cdc37, cell division cycle 37; CDK4, Cyclin-dependent kinase 4; CHOP, C/EBP homologous protein; cIAP, cellular inhibitor of apoptosis protein; CK2, casein kinase 2; c-PARP, cleaved poly(ADP-ribose) polymerase; COX-2, cyclooxygenase-2; Cyt *c*, cytochrome *c*; DR4, death receptors 4; DR5, death receptors 5; EGFR, epidermal growth factor receptor; Endo G, endonuclease G; ER- β, estrogen receptor β; ErbB2, c-Neu or human EGF receptor 2; ERK, extracellular signal-regulated protein kinases; Ezh2, enhancer of zeste homolog 2; FAK, focal adhesion kinase; Fas, apoptosis stimulating fragment; FOXO3a, forkhead box O3a; GPX4, glutathione peroxidase; GRP78, glucose regulatory protein 78; GSH, glutathione; GSK-3β, glycogen synthase kinase-3 beta; HIF-1α, hypoxia-inducible factor 1-alpha; Hsp90, heat shock protein 90; iNOS, inducible nitric oxide synthase; H2AX, histone H2A, X; HDAC, histone deacetylase; JAK, Janus family of tyrosine kinase; JAK2, Janus kinase 2; Kip1, Kinase inhibitory protein; IGF-IR, Type 1 insulin-like growth factor receptor; IKK β, Inhibitory kappa B kinase beta (IKKβ); IL-6, interleukin-6; LC3-I, clathrin light chain I; LC3-II, clathrin light chain II; LDH, lactate dehydrogenase; MAPK, mitogen-activated protein kinase; Mcl-1, myeloid cell leukemia-1; MLKL, mixed-lineage kinase domain-like pseudokinase; MMP, mitochondrial membrane potential; mTOR, mammalian target of rapamycin; PI3K, phosphoinositide 3-kinase; NAG-1, nonsteroidal anti-inflammatory drug (NSAID)-activated gene-1; NF-κB, nuclear factor kappa-light-chain-enhancer of activated B cells; Nrf2, nuclear factor erythroid 2-related factor 2; NQO2, NRH-quinone oxidoreductase 2; PERK, protein kinase RNA-like endoplasmic reticulum kinase; PKCδ, protein kinase C delta; PLK-1, Polo-like kinase 1; PTEN, Phosphatase and tensin homolog; RIP3, receptor-interacting protein 3; ROS, reactive oxygen species; SOD, superoxide dismutase; SQSTM1, Sequestosome 1; STAT, signal transducer and activator of transcription; TNFR, TNF receptor; TNF-α, tumor necrosis factor alpha; TRAIL, TNF-related apoptosis-inducing ligand; TRAIL-R, TRAIL receptor; ULK1, autophagy-activating kinase 1; VEGF, vascular endothelial growth factor; XIAP, X-linked inhibitor of apoptosis protein; YAP, yes-associated protein.

## Abbreviations

ABCG2	ATP-binding cassette subfamily G member 2
Akt	Protein kinase B
ALDH1	Acetaldehyde dehydrogenase 1
AMPK	AMP-activated protein kinase
ATF4	Activating transcription factor 4
ATM	Ataxia-telangiectasia mutated
Bad	Bcl-2-associated death promoter
Bak	Bcl-2 antagonist/killer
Bax	BCL2 associated X, apoptosis regulator
Bcl-2	B-cell lymphoma 2
Bim	Bcl-2 Interacting Mediator of cell death
BET	Bromodomain and extraterminal domain
BRD4	Bromodomain containing 4
CC	Colon cancer
CD	Cluster of differentiation
cdc	Cell division cycle
CDK	Cyclin-dependent kinase
CHOP	C/EBP homologous protein
CK2	Casein kinase 2
COX	Cyclooxygenase
CSCs	Cancer stem cells
CTGF	Connective tissue growth factor
CYR61	Cysteine-rich angiogenic inducer 61
DAPK	Death-associated protein kinase
DNA	Deoxyribonucleic Acid
DR	Death receptors
EGFR	Epidermal growth factor receptor
ECM	Extracellular matrix
EMT	Epithelial–mesenchymal transition
ERK	Extracellular signal-related kinase
EZH2	Enhancer of zeste homolog 2
FAKs	Focal adhesion kinases
FOX	Forkhead box
FTH1	Ferritin heavy chain 1
5-FU	5-fluorouracil
Gli1	GLI Family Zinc Finger 1
GSH	Glutathione
GPX4	Glutathione peroxidase 4
GSK-3β	Glycogen synthase kinase 3-beta
HCC	Hepatocellular carcinoma
HDAC	Histone deacetylases
HDM2	E3 ligase human double minute 2
HeLa	Human cervical carcinoma cells
HGF	Hepatocyte growth factor
HIF-1α	Hypoxia-inducible factor 1α
HSP	Heat shock protein 90
HUVECs	Human umbilical vein endothelial cells
IGF-IR	Type I insulin growth factor receptor
IL-6	Interleukin 6
IKKα	IκB kinase-α
IKK	IκB kinase
JAK	Janus kinase
JNK	Jun N-terminal kinase
LC3	Light chain 3
MAPK	Mitogen-activated protein kinase
Mcl-1	Myeloid cell leukemia-1
MEK	MAP kinase-ERK kinase
miRNAs	MicroRNA
MLKL	Mixed lineage kinase
MMPs	Metalloproteases
mRNA	Messenger ribonucleic acid
mTOR	Mammalian target of rapamycin
MVD	Microvessel density
NF-κB	Nuclear factor-kappa B
iNOS	Inducible nitric oxide synthase
NQO2	NRH-quinone oxidoreductase 2
Notch	Signal transducer and activator of transcription
NSCLC	Non-small cell lung carcinoma
Nrf2	Nuclear factor erythroid 2-related factor 2
PARP	Poly-ADP ribose polymerase
PCD	Programmed cell death
PCSC	Prostate cancer stem cells
PDGF-BB	Platelet-derived growth factor-BB
PDGFR-β	Platelet-derived growth factor receptor β
PERK	Protein kinase RNA-like endoplasmic reticulum kinase
PI3K	Phosphatidylinositol-3-kinase
PKB	Protein kinase B
PLK-1	Polo-like kinase 1
PTEN	Phosphatase and tensin homolog
PRRs	Pattern recognition receptors
p70S6K1	Ribosomal protein S6 kinase beta-1
RIPK3	Receptor-interacting serine/threonine kinase 3
ROS	Reactive oxygen species
SIRT	Sirtuin
Snail	Snail homolog 1/2 of drosophila
Sox2	SEX determining region (SRY) homology box 2
SPOCK1	Cwcv and kazal-like domains proteoglycan 1
pSTAT3	Phosphorylated signal transducer and activator of transcription-3
TAZ	PDZ-binding motif
TCRs	T cell receptors
TEADs	Transcriptional enhanced associate domain
hTERT	Human telomerase reverse transcriptase
TGF-β	Transforming growth factor-beta
TMZ	Temozolomide
TNBC	Triple-negative breast cancer
TNF	Tumor necrosis factor
TNFR	Tumor necrosis factor receptor
TRAIL	TNF-related apoptosis-inducing ligand
TWIST	Twist family bHLH transcription factor
ULK1	Kinase UNC51-like kinase-1
VEGF	Vascular endothelial growth factor
VEGFR2	Vascular endothelial growth factor receptor-2
YAP	Hippo-Yes-associated protein
ZEB1/2	Zinc finger E-box binding homeobox 1/2
ZO-1	Zonula Occludens-1

## References

[1] Ferlay J., Colombet M., Soerjomataram I., Parkin D.M., Piñeros M., Znaor A., Bray F. (2021). Cancer Statistics for the Year 2020: An Overview. Int. J. Cancer.

[2] Stoletov K., Beatty P.H., Lewis J.D. (2020). Novel Therapeutic Targets for Cancer Metastasis. Expert. Rev. Anticancer Ther..

[3] Gupta G.P., Massagué J. (2006). Cancer Metastasis: Building a Framework. Cell.

[4] Suhail Y., Cain M.P., Vanaja K., Kurywchak P.A., Levchenko A., Kalluri R. (2019). Kshitiz Systems Biology of Cancer Metastasis. Cell Syst..

[5] Babaei G., Aziz S.G.G., Jaghi N.Z.Z. (2021). EMT, Cancer Stem Cells and Autophagy; The Three Main Axes of Metastasis. Biomed. Pharmacother..

[6] Majidpoor J., Mortezaee K. (2021). Steps in Metastasis: An Updated Review. Med. Oncol..

[7] Hazafa A., Rehman K.U., Jahan N., Jabeen Z. (2020). The Role of Polyphenol (Flavonoids) Compounds in the Treatment of Cancer Cells. Nutr. Cancer.

[8] Kopustinskiene D.M., Jakstas V., Savickas A., Bernatoniene J. (2020). Flavonoids as Anticancer Agents. Nutrients.

[9] Rahmani A.H., Alsahli M.A., Almatroudi A., Almogbel M.A., Khan A.A., Anwar S., Almatroodi S.A. (2022). The Potential Role of Apigenin in Cancer Prevention and Treatment. Molecules.

[10] Nozhat Z., Heydarzadeh S., Memariani Z., Ahmadi A. (2021). Chemoprotective and Chemosensitizing Effects of Apigenin on Cancer Therapy. Cancer Cell Int..

[11] Javed Z., Sadia H., Iqbal M.J., Shamas S., Malik K., Ahmed R., Raza S., Butnariu M., Cruz-Martins N., Sharifi-Rad J. (2021). Apigenin Role as Cell-Signaling Pathways Modulator: Implications in Cancer Prevention and Treatment. Cancer Cell Int..

[12] Ashrafizadeh M., Bakhoda M.R., Bahmanpour Z., Ilkhani K., Zarrabi A., Makvandi P., Khan H., Mazaheri S., Darvish M., Mirzaei H. (2020). Apigenin as Tumor Suppressor in Cancers: Biotherapeutic Activity, Nanodelivery, and Mechanisms With Emphasis on Pancreatic Cancer. Front. Chem..

[13] Sung B., Chung H.Y., Kim N.D. (2016). Role of Apigenin in Cancer Prevention via the Induction of Apoptosis and Autophagy. J. Cancer Prev..

[14] Jäger A.K., Krydsfeldt K., Rasmussen H.B. (2009). Bioassay-Guided Isolation of Apigenin with GABA-Benzodiazepine Activity from Tanacetum Parthenium. Phytother. Res..

[15] Aslam Bhatti H., Noor R. (2019). Isolation of Apigenin by Solute-Solvent Extraction from Symphotrichum Novea Anglea. Integr. Food Nutr. Metab..

[16] Daneshvar S., Zamanian M.Y., Ivraghi M.S., Golmohammadi M., Modanloo M., Kamiab Z., Pourhosseini S.M.E., Heidari M., Bazmandegan G. (2023). A Comprehensive View on the Apigenin Impact on Colorectal Cancer: Focusing on Cellular and Molecular Mechanisms. Food Sci. Nutr..

[17] Wang M., Firrman J., Liu L.S., Yam K. (2019). A Review on Flavonoid Apigenin: Dietary Intake, ADME, Antimicrobial Effects, and Interactions with Human Gut Microbiota. BioMed Res. Int..

[18] Thomas S.D., Jha N.K., Jha S.K., Sadek B., Ojha S. (2023). Pharmacological and Molecular Insight on the Cardioprotective Role of Apigenin. Nutrients.

[19] Tang D., Chen K., Huang L., Li J. (2017). Pharmacokinetic Properties and Drug Interactions of Apigenin, a Natural Flavone. Expert. Opin. Drug Metab. Toxicol..

[20] Gradolatto A., Basly J.P., Berges R., Teyssier C., Chagnon M.C., Siess M.H., Canivenc-Lavier M.C. (2005). Pharmacokinetics and Metabolism of Apigenin in Female and Male Rats after a Single Oral Administration. Drug Metab. Dispos..

[21] Lee I.G., Lee J., Hong S.H., Seo Y.J. (2023). Apigenin’s Therapeutic Potential Against Viral Infection. Front. Biosci..

[22] Zhu L., Zhang H., Zhang X., Xia L., Zhang J.J. (2023). Research Progress on Antisepsis Effect of Apigenin and Its Mechanism of Action. Heliyon.

[23] Kashyap P., Shikha D., Thakur M., Aneja A. (2022). Functionality of Apigenin as a Potent Antioxidant with Emphasis on Bioavailability, Metabolism, Action Mechanism and in Vitro and in Vivo Studies: A Review. J. Food Biochem..

[24] Yan X., Qi M., Li P., Zhan Y., Shao H. (2017). Apigenin in Cancer Therapy: Anti-Cancer Effects and Mechanisms of Action. Cell Biosci..

[25] Liekens S., De Clercq E., Neyts J. (2001). Angiogenesis: Regulators and Clinical Applications. Biochem. Pharmacol..

[26] Unwith S., Zhao H., Hennah L., Ma D. (2015). The Potential Role of HIF on Tumour Progression and Dissemination. Int. J. Cancer.

[27] Krock B.L., Skuli N., Simon M.C. (2011). Hypoxia-Induced Angiogenesis: Good and Evil. Genes Cancer.

[28] Fu J., Zeng W., Chen M., Huang L., Li S., Li Z., Pan Q., Lv S., Yang X., Wang Y. (2022). Apigenin Suppresses Tumor Angiogenesis and Growth via Inhibiting HIF-1α Expression in Non-Small Cell Lung Carcinoma. Chem. Biol. Interact..

[29] Fang J., Xia C., Cao Z., Zheng J.Z., Reed E., Jiang B.-H. (2005). Apigenin Inhibits VEGF and HIF-1 Expression via PI3K/AKT/P70S6K1 and HDM2/P53 Pathways. FASEB J..

[30] Liu L.Z., Fang J., Zhou Q., Hu X., Shi X., Jiang B.H. (2005). Apigenin Inhibits Expression of Vascular Endothelial Growth Factor and Angiogenesis in Human Lung Cancer Cells: Implication of Chemoprevention of Lung Cancer. Mol. Pharmacol..

[31] Choi H.J., Eun J.S., Kim B.G., Kim S.Y., Jeon H., Soh Y. (2006). Vitexin, an HIF-1alpha Inhibitor, Has Anti-Metastatic Potential in PC12 Cells. Mol. Cells.

[32] Brabletz S., Schuhwerk H., Brabletz T., Stemmler M.P. (2021). Dynamic EMT: A Multi-Tool for Tumor Progression. EMBO J..

[33] Huang Y., Hong W., Wei X. (2022). The Molecular Mechanisms and Therapeutic Strategies of EMT in Tumor Progression and Metastasis. J. Hematol. Oncol..

[34] Akrida I., Bravou V., Papadaki H. (2022). The Deadly Cross-Talk between Hippo Pathway and Epithelial-Mesenchymal Transition (EMT) in Cancer. Mol. Biol. Rep..

[35] Kim D.H., Xing T., Yang Z., Dudek R., Lu Q., Chen Y.H. (2017). Epithelial Mesenchymal Transition in Embryonic Development, Tissue Repair and Cancer: A Comprehensive Overview. J. Clin. Med..

[36] Gonzalez D.M., Medici D. (2014). Signaling Mechanisms of the Epithelial-Mesenchymal Transition. Sci. Signal.

[37] Pastushenko I., Blanpain C. (2019). EMT Transition States during Tumor Progression and Metastasis. Trends Cell Biol..

[38] Du B., Shim J.S. (2016). Targeting Epithelial-Mesenchymal Transition (EMT) to Overcome Drug Resistance in Cancer. Molecules.

[39] Tong J., Shen Y., Zhang Z., Hu Y., Zhang X., Han L. (2019). Apigenin Inhibits Epithelial-Mesenchymal Transition of Human Colon Cancer Cells through NF-ΚB/Snail Signaling Pathway. Biosci. Rep..

[40] Qin Y., Zhao D., Zhou H.G., Wang X.H., Zhong W.L., Chen S., Gu W.G., Wang W., Zhang C.H., Liu Y.R. (2016). Apigenin Inhibits NF-ΚB and Snail Signaling, EMT and Metastasis in Human Hepatocellular Carcinoma. Oncotarget.

[41] Chien M.H., Lin Y.W., Wen Y.C., Yang Y.C., Hsiao M., Chang J.L., Huang H.C., Lee W.J. (2019). Targeting the SPOCK1-Snail/Slug Axis-Mediated Epithelial-to-Mesenchymal Transition by Apigenin Contributes to Repression of Prostate Cancer Metastasis. J. Exp. Clin. Cancer Res..

[42] Sun L.R., Li S.Y., Guo Q.S., Zhou W., Zhang H.M. (2020). SPOCK1 Involvement in Epithelial-to-Mesenchymal Transition: A New Target in Cancer Therapy?. Cancer Manag. Res..

[43] Chen D., Zhou H., Liu G., Zhao Y., Cao G., Liu Q. (2018). SPOCK1 Promotes the Invasion and Metastasis of Gastric Cancer through Slug-Induced Epithelial-Mesenchymal Transition. J. Cell Mol. Med..

[44] Chen Q., Yao Y.T., Xu H., Chen Y.B., Gu M., Cai Z.K., Wang Z. (2016). SPOCK1 Promotes Tumor Growth and Metastasis in Human Prostate Cancer. Drug Des. Devel. Ther..

[45] Chen Y.H., Wu J.X., Yang S.F., Yang C.K., Chen T.H., Hsiao Y.H. (2022). Anticancer Effects and Molecular Mechanisms of Apigenin in Cervical Cancer Cells. Cancers.

[46] Zhao X., Zhou H.B., Liu J., Xie J., Hu R. (2021). Apigenin Suppresses Proliferation, Invasion, and Epithelial-Mesenchymal Transition of Cervical Carcinoma Cells by Regulation of MiR-152/BRD4 Axis. Kaohsiung J. Med. Sci..

[47] Chang J.H., Cheng C.W., Yang Y.C., Chen W.S., Hung W.Y., Chow J.M., Chen P.S., Hsiao M., Lee W.J., Chien M.H. (2018). Downregulating CD26/DPPIV by Apigenin Modulates the Interplay between Akt and Snail/Slug Signaling to Restrain Metastasis of Lung Cancer with Multiple EGFR Statuses. J. Exp. Clin. Cancer Res..

[48] Lee H.H., Jung J., Moon A., Kang H., Cho H. (2019). Antitumor and Anti-Invasive Effect of Apigenin on Human Breast Carcinoma through Suppression of IL-6 Expression. Int. J. Mol. Sci..

[49] Cao H.H., Chu J.H., Kwan H.Y., Su T., Yu H., Cheng C.Y., Fu X.Q., Guo H., Li T., Tse A.K.W. (2016). Inhibition of the STAT3 Signaling Pathway Contributes to Apigenin-Mediated Anti-Metastatic Effect in Melanoma. Sci. Rep..

[50] García-García V.A., Alameda J.P., Page A., Mérida-García A., Navarro M., Tejero A., Paramio J.M., García-Fernández R.A., Casanova M.L. (2022). IKKα Induces Epithelial-Mesenchymal Changes in Mouse Skin Carcinoma Cells That Can Be Partially Reversed by Apigenin. Int. J. Mol. Sci..

[51] Ganesan K., Xu B. (2017). Molecular Targets of Vitexin and Isovitexin in Cancer Therapy: A Critical Review. Ann. N. Y. Acad. Sci..

[52] Zhu H., Zhao N., Jiang M. (2021). Isovitexin Attenuates Tumor Growth in Human Colon Cancer Cells through the Modulation of Apoptosis and Epithelial-Mesenchymal Transition via PI3K/Akt/MTOR Signaling Pathway. Biochem. Cell Biol..

[53] Atashzar M.R., Baharlou R., Karami J., Abdollahi H., Rezaei R., Pourramezan F., Zoljalali Moghaddam S.H. (2020). Cancer Stem Cells: A Review from Origin to Therapeutic Implications. J. Cell Physiol..

[54] Tong W.W., Tong G.H., Liu Y. (2018). Cancer Stem Cells and Hypoxia-Inducible Factors (Review). Int. J. Oncol..

[55] Peng F., Liao M., Qin R., Zhu S., Peng C., Fu L., Chen Y., Han B. (2022). Regulated Cell Death (RCD) in Cancer: Key Pathways and Targeted Therapies. Signal Transduct. Target. Ther..

[56] Walcher L., Kistenmacher A.K., Suo H., Kitte R., Dluczek S., Strauß A., Blaudszun A.R., Yevsa T., Fricke S., Kossatz-Boehlert U. (2020). Cancer Stem Cells-Origins and Biomarkers: Perspectives for Targeted Personalized Therapies. Front. Immunol..

[57] Carnero A., Garcia-Mayea Y., Mir C., Lorente J., Rubio I.T., LLeonart M.E. (2016). The Cancer Stem-Cell Signaling Network and Resistance to Therapy. Cancer Treat. Rev..

[58] Yang L., Shi P., Zhao G., Xu J., Peng W., Zhang J., Zhang G., Wang X., Dong Z., Chen F. (2020). Targeting Cancer Stem Cell Pathways for Cancer Therapy. Signal Transduct. Target. Ther..

[59] Zhang Y., Wang X. (2020). Targeting the Wnt/β-Catenin Signaling Pathway in Cancer. J. Hematol. Oncol..

[60] Lian I., Kim J., Okazawa H., Zhao J., Zhao B., Yu J., Chinnaiyan A., Israel M.A., Goldstein L.S.B., Abujarour R. (2010). The Role of YAP Transcription Coactivator in Regulating Stem Cell Self-Renewal and Differentiation. Genes Dev..

[61] Ajani J.A., Song S., Hochster H.S., Steinberg I.B. (2015). Cancer Stem Cells: The Promise and the Potential. Semin. Oncol..

[62] Ishiguro T., Ohata H., Sato A., Yamawaki K., Enomoto T., Okamoto K. (2017). Tumor-Derived Spheroids: Relevance to Cancer Stem Cells and Clinical Applications. Cancer Sci..

[63] Yousefnia S., Ghaedi K., Seyed Forootan F., Nasr Esfahani M.H. (2019). Characterization of the Stemness Potency of Mammospheres Isolated from the Breast Cancer Cell Lines. Tumour Biol..

[64] Dandawate P.R., Subramaniam D., Jensen R.A., Anant S. (2016). Targeting Cancer Stem Cells and Signaling Pathways by Phytochemicals: Novel Approach for Breast Cancer Therapy. Semin. Cancer Biol..

[65] Gu H.F., Mao X.Y., Du M. (2020). Prevention of Breast Cancer by Dietary Polyphenols-Role of Cancer Stem Cells. Crit. Rev. Food Sci. Nutr..

[66] Ghanbari-Movahed M., Shafiee S., Burcher J.T., Lagoa R., Farzaei M.H., Bishayee A. (2023). Anticancer Potential of Apigenin and Isovitexin with Focus on Oncogenic Metabolism in Cancer Stem Cells. Metabolites.

[67] Kim B., Jung N., Lee S., Sohng J.K., Jung H.J. (2016). Apigenin Inhibits Cancer Stem Cell-Like Phenotypes in Human Glioblastoma Cells via Suppression of c-Met Signaling. Phytother. Res..

[68] Hong W., Guan K.L. (2012). The YAP and TAZ Transcription Co-Activators: Key Downstream Effectors of the Mammalian Hippo Pathway. Semin. Cell Dev. Biol..

[69] Li Y.W., Xu J., Zhu G.Y., Huang Z.J., Lu Y., Li X.Q., Wang N., Zhang F.X. (2018). Apigenin Suppresses the Stem Cell-like Properties of Triple-Negative Breast Cancer Cells by Inhibiting YAP/TAZ Activity. Cell Death Discov..

[70] Sharma A., Sinha S., Keswani H., Shrivastava N. (2022). Kaempferol and Apigenin Suppresses the Stemness Properties of TNBC Cells by Modulating Sirtuins. Mol. Divers..

[71] Sharpe B., Beresford M., Bowen R., Mitchard J., Chalmers A.D. (2013). Searching for Prostate Cancer Stem Cells: Markers and Methods. Stem Cell Rev. Rep..

[72] Erdogan S., Doganlar O., Doganlar Z.B., Serttas R., Turkekul K., Dibirdik I., Bilir A. (2016). The Flavonoid Apigenin Reduces Prostate Cancer CD44(+) Stem Cell Survival and Migration through PI3K/Akt/NF-ΚB Signaling. Life Sci..

[73] Liang C., Jiang Y., Sun L. (2023). Vitexin Suppresses the Proliferation, Angiogenesis and Stemness of Endometrial Cancer through the PI3K/AKT Pathway. Pharm. Biol..

[74] Firnau M.B., Brieger A. (2022). CK2 and the Hallmarks of Cancer. Biomedicines.

[75] Zhang S., Wang Y., Mao J.H., Hsieh D., Kim I.J., Hu L.M., Xu Z., Long H., Jablons D.M., You L. (2012). Inhibition of CK2α Down-Regulates Hedgehog/Gli Signaling Leading to a Reduction of a Stem-like Side Population in Human Lung Cancer Cells. PLoS ONE.

[76] Tang A.Q., Cao X.C., Tian L., He L., Liu F. (2015). Apigenin Inhibits the Self-Renewal Capacity of Human Ovarian Cancer SKOV3-derived Sphere-Forming Cells. Mol. Med. Rep..

[77] Liu J., Cao X.C., Xiao Q., Quan M.F. (2015). Apigenin Inhibits HeLa Sphere-Forming Cells through Inactivation of Casein Kinase 2α. Mol. Med. Rep..

[78] Ketkaew Y., Osathanon T., Pavasant P., Sooampon S. (2017). Apigenin Inhibited Hypoxia Induced Stem Cell Marker Expression in a Head and Neck Squamous Cell Carcinoma Cell Line. Arch. Oral. Biol..

[79] Xu C., Cao X., Cao X., Liu L., Qiu Y., Li X., Zhou L., Ning Y., Ren K., Cao J. (2020). Isovitexin Inhibits Stemness and Induces Apoptosis in Hepatocellular Carcinoma SK-Hep-1 Spheroids by Upregulating MiR-34a Expression. Anticancer Agents Med. Chem..

[80] Huang T., Alvarez A., Hu B., Cheng S.Y. (2013). Noncoding RNAs in Cancer and Cancer Stem Cells. Chin. J. Cancer.

[81] Li Y., Chen X., He W., Xia S., Jiang X., Li X., Bai J., Li N., Chen L., Yang B. (2021). Apigenin Enhanced Antitumor Effect of Cisplatin in Lung Cancer via Inhibition of Cancer Stem Cells. Nutr. Cancer.

[82] Erdogan S., Turkekul K., Serttas R., Erdogan Z. (2017). The Natural Flavonoid Apigenin Sensitizes Human CD44+ Prostate Cancer Stem Cells to Cisplatin Therapy. Biomed. Pharmacother..

[83] Zhang X., Zhang W., Chen F., Lu Z. (2021). Combined Effect of Chrysin and Apigenin on Inhibiting the Development and Progression of Colorectal Cancer by Suppressing the Activity of P38-MAPK/AKT Pathway. IUBMB Life.

[84] Farghadani R., Naidu R. (2023). The Anticancer Mechanism of Action of Selected Polyphenols in Triple-Negative Breast Cancer (TNBC). Biomed. Pharmacother..

[85] Abotaleb M., Samuel S.M., Varghese E., Varghese S., Kubatka P., Liskova A., Büsselberg D. (2018). Flavonoids in Cancer and Apoptosis. Cancers.

[86] Chen Y., Wang S., Geng B., Yi Z. (2018). Pelargonidin Induces Antitumor Effects in Human Osteosarcoma Cells via Autophagy Induction, Loss of Mitochondrial Membrane Potential, G2/M Cell Cycle Arrest and Downregulation of PI3K/AKT Signalling Pathway. J. BUON.

[87] Dash B.C., El-Deiry W.S. (2005). Phosphorylation of P21 in G2/M Promotes Cyclin B-Cdc2 Kinase Activity. Mol. Cell Biol..

[88] Ittiudomrak T., Puthong S., Roytrakul S., Chanchao C. (2019). α-Mangostin and Apigenin Induced Cell Cycle Arrest and Programmed Cell Death in SKOV-3 Ovarian Cancer Cells. Toxicol. Res..

[89] Lee Y., Sung B., Kang Y.J., Kim D.H., Jang J.Y., Hwang S.Y., Kim M., Lim H.S., Yoon J.H., Chung H.Y. (2014). Apigenin-Induced Apoptosis Is Enhanced by Inhibition of Autophagy Formation in HCT116 Human Colon Cancer Cells. Int. J. Oncol..

[90] Zhang L., Cheng X., Gao Y., Zheng J., Xu Q., Sun Y., Guan H., Yu H., Sun Z. (2015). Apigenin Induces Autophagic Cell Death in Human Papillary Thyroid Carcinoma BCPAP Cells. Food Funct..

[91] Ujiki M.B., Ding X.Z., Salabat M.R., Bentrem D.J., Golkar L., Milam B., Talamonti M.S., Bell R.H., Iwamura T., Adrian T.E. (2006). Apigenin Inhibits Pancreatic Cancer Cell Proliferation through G2/M Cell Cycle Arrest. Mol. Cancer.

[92] Lepley D.M., Li B., Birt D.F., Pelling J.C. (1996). The Chemopreventive Flavonoid Apigenin Induces G2/M Arrest in Keratinocytes. Carcinogenesis.

[93] O’Prey J., Brown J., Fleming J., Harrison P.R. (2003). Effects of Dietary Flavonoids on Major Signal Transduction Pathways in Human Epithelial Cells. Biochem. Pharmacol..

[94] Casagrande F., Darbon J.M. (2001). Effects of Structurally Related Flavonoids on Cell Cycle Progression of Human Melanoma Cells: Regulation of Cyclin-Dependent Kinases CDK2 and CDK1. Biochem. Pharmacol..

[95] McVean M., Weinberg W.C., Pelling J.C. (2002). A P21(Waf1)-Independent Pathway for Inhibitory Phosphorylation of Cyclin-Dependent Kinase P34(Cdc2) and Concomitant G(2)/M Arrest by the Chemopreventive Flavonoid Apigenin. Mol. Carcinog..

[96] Gupta S., Afaq F., Mukhtar H. (2001). Selective Growth-Inhibitory, Cell-Cycle Deregulatory and Apoptotic Response of Apigenin in Normal versus Human Prostate Carcinoma Cells. Biochem. Biophys. Res. Commun..

[97] Wang W., Heideman L., Chung C.S., Pelling J.C., Koehler K.J., Birt D.F. (2000). Cell-Cycle Arrest at G2/M and Growth Inhibition by Apigenin in Human Colon Carcinoma Cell Lines. Mol. Carcinog..

[98] Zhao G., Han X., Cheng W., Ni J., Zhang Y., Lin J., Song Z. (2017). Apigenin Inhibits Proliferation and Invasion, and Induces Apoptosis and Cell Cycle Arrest in Human Melanoma Cells. Oncol. Rep..

[99] Tseng T.H., Chien M.H., Lin W.L., Wen Y.C., Chow J.M., Chen C.K., Kuo T.C., Lee W.J. (2017). Inhibition of MDA-MB-231 Breast Cancer Cell Proliferation and Tumor Growth by Apigenin through Induction of G2/M Arrest and Histone H3 Acetylation-Mediated P21WAF1/CIP1 Expression. Environ. Toxicol..

[100] Shendge A.K., Chaudhuri D., Mandal N. (2021). The Natural Flavones, Acacetin and Apigenin, Induce Cdk-Cyclin Mediated G2/M Phase Arrest and Trigger ROS-Mediated Apoptosis in Glioblastoma Cells. Mol. Biol. Rep..

[101] Der Shi M., Shiao C.K., Lee Y.C., Shih Y.W. (2015). Apigenin, a Dietary Flavonoid, Inhibits Proliferation of Human Bladder Cancer T-24 Cells via Blocking Cell Cycle Progression and Inducing Apoptosis. Cancer Cell Int..

[102] Zhu Y., Mao Y., Chen H., Lin Y., Hu Z., Wu J., Xu X., Xu X., Qin J., Xie L. (2013). Apigenin Promotes Apoptosis, Inhibits Invasion and Induces Cell Cycle Arrest of T24 Human Bladder Cancer Cells. Cancer Cell Int..

[103] Kim N.W., Piatyszek M.A., Prowse K.R., Harley C.B., West M.D., Ho P.L.C., Coviello G.M., Wright W.E., Weinrich S.L., Shay J.W. (1994). Specific Association of Human Telomerase Activity with Immortal Cells and Cancer. Science.

[104] Cabuy E., De Ridder L. (2001). Telomerase Activity and Expression of Telomerase Reverse Transcriptase Correlated with Cell Proliferation in Meningiomas and Malignant Brain Tumors in Vivo. Virchows Arch..

[105] Chakrabarti M., Banik N.L., Ray S.K. (2013). Sequential HTERT Knockdown and Apigenin Treatment Inhibited Invasion and Proliferation and Induced Apoptosis in Human Malignant Neuroblastoma SK-N-DZ and SK-N-BE2 Cells. J. Mol. Neurosci..

[106] Lepley D.M., Pelling J.C. (1997). Induction of P21/WAF1 and G1 Cell-Cycle Arrest by the Chemopreventive Agent Apigenin. Mol. Carcinog..

[107] Zheng P.W., Chiang L.C., Lin C.C. (2005). Apigenin Induced Apoptosis through P53-Dependent Pathway in Human Cervical Carcinoma Cells. Life Sci..

[108] Wang D., Wang Z., Dai X., Zhang L., Li M. (2024). Apigenin and Temozolomide Synergistically Inhibit Glioma Growth Through the PI3K/ AKT Pathway. Cancer Biother. Radiopharm..

[109] Park J.H., Pyun W.Y., Park H.W. (2020). Cancer Metabolism: Phenotype, Signaling and Therapeutic Targets. Cells.

[110] Lee E., Song C.H., Bae S.J., Ha K.T., Karki R. (2023). Regulated Cell Death Pathways and Their Roles in Homeostasis, Infection, Inflammation, and Tumorigenesis. Exp. Mol. Med..

[111] He S., Huang Q., Cheng J. (2023). The Unfolding Story of Dying Tumor Cells during Cancer Treatment. Front. Immunol..

[112] Carneiro B.A., El-Deiry W.S. (2020). Targeting Apoptosis in Cancer Therapy. Nat. Rev. Clin. Oncol..

[113] Nonnenmacher L., Hasslacher S., Zimmermann J., Karpel-Massler G., La Ferla-Brühl K., Barry S.E., Burster T., Siegelin M.D., Brühl O., Halatsch M.E. (2016). Cell Death Induction in Cancer Therapy—Past, Present, and Future. Crit. Rev. Oncog..

[114] Amini P., Moazamiyanfar R., Dakkali M.S., Jafarzadeh E., Ganjizadeh M., Rastegar-Pouyani N., Moloudi K., Khodamoradi E., Taeb S., Najafi M. (2023). Induction of Cancer Cell Death by Apigenin: A Review on Different Cell Death Pathways. Mini Rev. Med. Chem..

[115] Jang J.Y., Sung B., Kim N.D. (2022). Role of Induced Programmed Cell Death in the Chemopreventive Potential of Apigenin. Int. J. Mol. Sci..

[116] Kashyap D., Garg V.K., Goel N. (2021). Intrinsic and Extrinsic Pathways of Apoptosis: Role in Cancer Development and Prognosis. Adv. Protein Chem. Struct. Biol..

[117] Kilani-Jaziri S., Frachet V., Bhouri W., Ghedira K., Chekir-Ghedira L., Ronot X. (2012). Flavones Inhibit the Proliferation of Human Tumor Cancer Cell Lines by Inducing Apoptosis. Drug Chem. Toxicol..

[118] Bahreghani M.T., Geraily G., Alizadeh S., Najafi M., Shirazi A. (2021). Apigenin Enhanced Radiation-Induced Apoptosis/Necrosis by Sensitization of LNCaP Prostate Cancer Cells to 6 MV Photon Beams. Cell J..

[119] Lin C.H., Chang C.Y., Lee K.R., Lin H.J., Chen T.H., Wan L. (2015). Flavones Inhibit Breast Cancer Proliferation through the Akt/FOXO3a Signaling Pathway. BMC Cancer.

[120] Johnson J.L., De Mejia E.G. (2013). Flavonoid Apigenin Modified Gene Expression Associated with Inflammation and Cancer and Induced Apoptosis in Human Pancreatic Cancer Cells through Inhibition of GSK-3β/NF-ΚB Signaling Cascade. Mol. Nutr. Food Res..

[121] Lu H.F., Chie Y.U.J., Yang M.S., Lee C.S., Fu J.J., Yang J.S., Tan T.W., Wu S.H., Ma Y.I.S., Ip S.W. (2010). Apigenin Induces Caspase-Dependent Apoptosis in Human Lung Cancer A549 Cells through Bax- and Bcl-2-Triggered Mitochondrial Pathway. Int. J. Oncol..

[122] Bruno A., Siena L., Gerbino S., Ferraro M., Chanez P., Giammanco M., Gjomarkaj M., Pace E. (2011). Apigenin Affects Leptin/Leptin Receptor Pathway and Induces Cell Apoptosis in Lung Adenocarcinoma Cell Line. Eur. J. Cancer.

[123] Lu H.F., Chie Y.J., Yang M.S., Lu K.W., Fu J.J., Yang J.S., Chen H.Y., Hsia T.C., Ma C.Y., Ip S.W. (2011). Apigenin Induces Apoptosis in Human Lung Cancer H460 Cells through Caspase- and Mitochondria-Dependent Pathways. Hum. Exp. Toxicol..

[124] Chen J., Chen J., Li Z., Liu C., Yin L. (2014). The Apoptotic Effect of Apigenin on Human Gastric Carcinoma Cells through Mitochondrial Signal Pathway. Tumour Biol..

[125] Shukla S., Fu P., Gupta S. (2014). Apigenin Induces Apoptosis by Targeting Inhibitor of Apoptosis Proteins and Ku70-Bax Interaction in Prostate Cancer. Apoptosis.

[126] Der Way T., Kao M.C., Lin J.K. (2005). Degradation of HER2/Neu by Apigenin Induces Apoptosis through Cytochrome c Release and Caspase-3 Activation in HER2/Neu-Overexpressing Breast Cancer Cells. FEBS Lett..

[127] Cao X., Liu B., Cao W., Zhang W., Zhang F., Zhao H., Meng R., Zhang L., Niu R., Hao X. (2013). Autophagy Inhibition Enhances Apigenin-Induced Apoptosis in Human Breast Cancer Cells. Chin. J. Cancer Res..

[128] Chen X., Xu H., Yu X., Wang X., Zhu X., Xu X. (2019). Apigenin Inhibits in Vitro and in Vivo Tumorigenesis in Cisplatin-Resistant Colon Cancer Cells by Inducing Autophagy, Programmed Cell Death and Targeting m-TOR/PI3K/Akt Signalling Pathway. JBUON.

[129] Turktekin M., Konac E., Onen H.I., Alp E., Yilmaz A., Menevse S. (2011). Evaluation of the Effects of the Flavonoid Apigenin on Apoptotic Pathway Gene Expression on the Colon Cancer Cell Line (HT29). J. Med. Food.

[130] Borah S.M., Kma L., Darjee M.S., Deka D., Lyngdoh A., Sharan R.N., Baruah T.J. (2023). Apigenin Promotes Cell Death in NCI-H23 Cells by Upregulation of PTEN: Potential Involvement of the Binding of Apigenin with WWP2 Protein. J. Biomol. Struct. Dyn..

[131] Huang S., Yu M., Shi N., Zhou Y., Li F., Li X., Huang X., Jin J. (2020). Apigenin and Abivertinib, a Novel BTK Inhibitor Synergize to Inhibit Diffuse Large B-Cell Lymphoma in Vivo and Vitro. J. Cancer.

[132] Kaur P., Shukla S., Gupta S. (2008). Plant Flavonoid Apigenin Inactivates Akt to Trigger Apoptosis in Human Prostate Cancer: An in Vitro and in Vivo Study. Carcinogenesis.

[133] Babcook M.A., Gupta S. Apigenin Modulates Insulin-like Growth Factor Axis: Implications for Prevention and Therapy of Prostate Cancer. Curr. Drug Targets.

[134] Shukla S., Gupta S. (2009). Apigenin Suppresses Insulin-like Growth Factor I Receptor Signaling in Human Prostate Cancer: An in Vitro and in Vivo Study. Mol. Carcinog..

[135] Johnstone R.W., Frew A.J., Smyth M.J. (2008). The TRAIL Apoptotic Pathway in Cancer Onset, Progression and Therapy. Nat. Rev. Cancer.

[136] Pimentel J.M., Zhou J.Y., Wu G.S. (2023). The Role of TRAIL in Apoptosis and Immunosurveillance in Cancer. Cancers.

[137] Khan T.H., Sultana S. (2006). Apigenin Induces Apoptosis in Hep G2 Cells: Possible Role of TNF-Alpha and IFN-Gamma. Toxicology.

[138] Morrissey C., O’Neill A., Spengler B., Christoffel V., Fitzpatrick J.M., Watson R.W.G. (2005). Apigenin Drives the Production of Reactive Oxygen Species and Initiates a Mitochondrial Mediated Cell Death Pathway in Prostate Epithelial Cells. Prostate.

[139] Oishi M., Iizumi Y., Taniguchi T., Goi W., Miki T., Sakai T. (2013). Apigenin Sensitizes Prostate Cancer Cells to Apo2L/TRAIL by Targeting Adenine Nucleotide Translocase-2. PLoS ONE.

[140] Chen M., Wang X., Zha D., Cai F., Zhang W., He Y., Huang Q., Zhuang H., Hua Z.C. (2016). Apigenin Potentiates TRAIL Therapy of Non-Small Cell Lung Cancer via Upregulating DR4/DR5 Expression in a P53-Dependent Manner. Sci. Rep..

[141] Kim E.Y., Kim A.K. (2012). Apigenin Sensitizes Huh-7 Human Hepatocellular Carcinoma Cells to TRAIL-Induced Apoptosis. Biomol. Ther..

[142] Kim E.Y., Yu J.S., Yang M., Kim A.K. (2013). Sub-Toxic Dose of Apigenin Sensitizes HepG2 Cells to TRAIL through ERK-Dependent up-Regulation of TRAIL Receptor DR5. Mol. Cells.

[143] Kang C.H., Molagoda I.M.N., Choi Y.H., Park C., Moon D.O., Kim G.Y. (2018). Apigenin Promotes TRAIL-Mediated Apoptosis Regardless of ROS Generation. Food Chem. Toxicol..

[144] Masuelli L., Marzocchella L., Quaranta A., Palumbo C., Pompa G., Izzi V., Canini A., Modesti A., Galvano F., Bei R. (2011). Apigenin Induces Apoptosis and Impairs Head and Neck Carcinomas EGFR/ErbB2 Signaling. Front. Biosci..

[145] Chan L.P., Chou T.H., Ding H.Y., Chen P.R., Chiang F.Y., Kuo P.L., Liang C.H. (2012). Apigenin Induces Apoptosis via Tumor Necrosis Factor Receptor- and Bcl-2-Mediated Pathway and Enhances Susceptibility of Head and Neck Squamous Cell Carcinoma to 5-Fluorouracil and Cisplatin. Biochim. Biophys. Acta.

[146] Stump T.A., Santee B.N., Williams L.P., Kunze R.A., Heinze C.E., Huseman E.D., Gryka R.J., Simpson D.S., Amos S. (2017). The Antiproliferative and Apoptotic Effects of Apigenin on Glioblastoma Cells. J. Pharm. Pharmacol..

[147] Ayele T.M., Muche Z.T., Teklemariam A.B., Kassie A.B., Abebe E.C. (2022). Role of JAK2/STAT3 Signaling Pathway in the Tumorigenesis, Chemotherapy Resistance, and Treatment of Solid Tumors: A Systemic Review. J. Inflamm. Res..

[148] Seo H.S., Jo J.K., Ku J.M., Choi H.S., Choi Y.K., Woo J.K., Kim H.I., Kang S.Y., Lee K.M., Nam K.W. (2015). Induction of Caspase-Dependent Extrinsic Apoptosis by Apigenin through Inhibition of Signal Transducer and Activator of Transcription 3 (STAT3) Signalling in HER2-Overexpressing BT-474 Breast Cancer Cells. Biosci. Rep..

[149] Seo H.S., Ku J.M., Choi H.S., Woo J.K., Jang B.H., Go H., Shin Y.C., Ko S.G. (2015). Apigenin Induces Caspase-Dependent Apoptosis by Inhibiting Signal Transducer and Activator of Transcription 3 Signaling in HER2-Overexpressing SKBR3 Breast Cancer Cells. Mol. Med. Rep..

[150] Seo H.S., Ku J.M., Choi H.S., Woo J.K., Jang B.H., Shin Y.C., Ko S.G. (2014). Induction of Caspase-Dependent Apoptosis by Apigenin by Inhibiting STAT3 Signaling in HER2-Overexpressing MDA-MB-453 Breast Cancer Cells. Anticancer Res..

[151] Seo H.S., Choi H.S., Kim S.R., Choi Y.K., Woo S.M., Shin I., Woo J.K., Park S.Y., Shin Y.C., Ko S.K. (2012). Apigenin Induces Apoptosis via Extrinsic Pathway, Inducing P53 and Inhibiting STAT3 and NFκB Signaling in HER2-Overexpressing Breast Cancer Cells. Mol. Cell Biochem..

[152] Choi E.J., Kim G.H. (2009). Apigenin Causes G(2)/M Arrest Associated with the Modulation of P21(Cip1) and Cdc2 and Activates P53-Dependent Apoptosis Pathway in Human Breast Cancer SK-BR-3 Cells. J. Nutr. Biochem..

[153] Maeda Y., Takahashi H., Nakai N., Yanagita T., Ando N., Okubo T., Saito K., Shiga K., Hirokawa T., Hara M. (2018). Apigenin Induces Apoptosis by Suppressing Bcl-Xl and Mcl-1 Simultaneously via Signal Transducer and Activator of Transcription 3 Signaling in Colon Cancer. Int. J. Oncol..

[154] Mohan N., Ai W., Chakrabarti M., Banik N.L., Ray S.K. (2013). KLF4 Overexpression and Apigenin Treatment down Regulated Anti-Apoptotic Bcl-2 Proteins and Matrix Metalloproteinases to Control Growth of Human Malignant Neuroblastoma SK-N-DZ and IMR-32 Cells. Mol. Oncol..

[155] Yu H., Liu Z., Zhou H., Dai W., Chen S., Shu Y., Feng J. (2012). JAK-STAT Pathway Modulates the Roles of INOS and COX-2 in the Cytoprotection of Early Phase of Hydrogen Peroxide Preconditioning against Apoptosis Induced by Oxidative Stress. Neurosci. Lett..

[156] Ruela-De-Sousa R.R., Fuhler G.M., Blom N., Ferreira C.V., Aoyama H., Peppelenbosch M.P. (2010). Cytotoxicity of Apigenin on Leukemia Cell Lines: Implications for Prevention and Therapy. Cell Death Dis..

[157] Budhraja A., Gao N., Zhang Z., Son Y.O., Cheng S., Wang X., Ding S., Hitron A., Chen G., Luo J. (2012). Apigenin Induces Apoptosis in Human Leukemia Cells and Exhibits Anti-Leukemic Activity in Vivo. Mol. Cancer Ther..

[158] Adham A.N., Abdelfatah S., Naqishbandi A.M., Mahmoud N., Efferth T. (2021). Cytotoxicity of Apigenin toward Multiple Myeloma Cell Lines and Suppression of INOS and COX-2 Expression in STAT1-Transfected HEK293 Cells. Phytomedicine.

[159] Arfin S., Jha N.K., Jha S.K., Kesari K.K., Ruokolainen J., Roychoudhury S., Rathi B., Kumar D. (2021). Oxidative Stress in Cancer Cell Metabolism. Antioxidants.

[160] Bai H., Jin H., Yang F., Zhu H., Cai J. (2014). Apigenin Induced MCF-7 Cell Apoptosis-Associated Reactive Oxygen Species. Scanning.

[161] Shendge A.K., Chaudhuri D., Basu T., Mandal N. (2021). A Natural Flavonoid, Apigenin Isolated from Clerodendrum Viscosum Leaves, Induces G2/M Phase Cell Cycle Arrest and Apoptosis in MCF-7 Cells through the Regulation of P53 and Caspase-Cascade Pathway. Clin. Transl. Oncol..

[162] Souza R.P., Bonfim-Mendonça P.D.S., Gimenes F., Ratti B.A., Kaplum V., Bruschi M.L., Nakamura C.V., Silva S.O., Maria-Engler S.S., Consolaro M.E.L. (2017). Oxidative Stress Triggered by Apigenin Induces Apoptosis in a Comprehensive Panel of Human Cervical Cancer-Derived Cell Lines. Oxid. Med. Cell Longev..

[163] Das S., Das J., Samadder A., Boujedaini N., Khuda-Bukhsh A.R. (2012). Apigenin-Induced Apoptosis in A375 and A549 Cells through Selective Action and Dysfunction of Mitochondria. Exp. Biol. Med..

[164] Shukla S., Gupta S. (2008). Apigenin-Induced Prostate Cancer Cell Death Is Initiated by Reactive Oxygen Species and P53 Activation. Free Radic. Biol. Med..

[165] Valdameri G., Trombetta-Lima M., Worfel P.R., Pires A.R.A., Martinez G.R., Noleto G.R., Cadena S.M.S.C., Sogayar M.C., Winnischofer S.M.B., Rocha M.E.M. (2011). Involvement of Catalase in the Apoptotic Mechanism Induced by Apigenin in HepG2 Human Hepatoma Cells. Chem. Biol. Interact..

[166] Zhang C., Liao Y., Li T., Zhong H., Shan L., Yu P., Xia C., Xu L. (2024). Apigenin Promotes Apoptosis of 4T1 Cells through PI3K/AKT/Nrf2 Pathway and Improves Tumor Immune Microenvironment in Vivo. Toxicol. Res..

[167] Juan C.A., de la Lastra J.M.P., Plou F.J., Pérez-Lebeña E. (2021). The Chemistry of Reactive Oxygen Species (ROS) Revisited: Outlining Their Role in Biological Macromolecules (DNA, Lipids and Proteins) and Induced Pathologies. Int. J. Mol. Sci..

[168] Arango D., Parihar A., Villamena F.A., Wang L., Freitas M.A., Grotewold E., Doseff A.I. (2012). Apigenin Induces DNA Damage through the PKCδ-Dependent Activation of ATM and H2AX Causing down-Regulation of Genes Involved in Cell Cycle Control and DNA Repair. Biochem. Pharmacol..

[169] Zhong Y., Krisanapun C., Lee S.H., Nualsanit T., Sams C., Peungvicha P., Baek S.J. (2010). Molecular Targets of Apigenin in Colorectal Cancer Cells: Involvement of P21, NAG-1 and P53. Eur. J. Cancer.

[170] Vargo M.A., Voss O.H., Poustka F., Cardounel A.J., Grotewold E., Doseff A.I. (2006). Apigenin-Induced-Apoptosis Is Mediated by the Activation of PKCdelta and Caspases in Leukemia Cells. Biochem. Pharmacol..

[171] Wang H., Guo M., Wei H., Chen Y. (2023). Targeting P53 Pathways: Mechanisms, Structures, and Advances in Therapy. Signal Transduct. Target. Ther..

[172] Cheung E.C., Vousden K.H. (2022). The Role of ROS in Tumour Development and Progression. Nat. Rev. Cancer.

[173] Zhang Q., Zhao X.H., Wang Z.J. (2008). Flavones and Flavonols Exert Cytotoxic Effects on a Human Oesophageal Adenocarcinoma Cell Line (OE33) by Causing G2/M Arrest and Inducing Apoptosis. Food Chem. Toxicol..

[174] Zhang Q., Zhao X.H., Wang Z.J. (2009). Cytotoxicity of Flavones and Flavonols to a Human Esophageal Squamous Cell Carcinoma Cell Line (KYSE-510) by Induction of G2/M Arrest and Apoptosis. Toxicol. In Vitro.

[175] Masuelli L., Benvenuto M., Mattera R., Di Stefano E., Zago E., Taffera G., Tresoldi I., Giganti M.G., Frajese G.V., Berardi G. (2017). In Vitro and In Vivo Anti-Tumoral Effects of the Flavonoid Apigenin in Malignant Mesothelioma. Front. Pharmacol..

[176] Eun J.C., Kim G.H. (2009). Apigenin Induces Apoptosis through a Mitochondria/Caspase-Pathway in Human Breast Cancer MDA-MB-453 Cells. J. Clin. Biochem. Nutr..

[177] Torkin R., Lavoie J.F., Kaplan D.R., Yeger H. (2005). Induction of Caspase-Dependent, P53-Mediated Apoptosis by Apigenin in Human Neuroblastoma. Mol. Cancer Ther..

[178] Meng S., Zhu Y., Li J.F., Wang X., Liang Z., Li S.Q., Xu X., Chen H., Liu B., Zheng X.Y. (2017). Apigenin Inhibits Renal Cell Carcinoma Cell Proliferation. Oncotarget.

[179] Abraham A.G., O’Neill E. (2014). PI3K/Akt-Mediated Regulation of P53 in Cancer. Biochem. Soc. Trans..

[180] Granato M., Gilardini Montani M.S., Santarelli R., D’Orazi G., Faggioni A., Cirone M. (2017). Apigenin, by Activating P53 and Inhibiting STAT3, Modulates the Balance between pro-Apoptotic and pro-Survival Pathways to Induce PEL Cell Death. J. Exp. Clin. Cancer Res..

[181] Kim S.H., Kang J.G., Kim C.S., Ihm S.H., Choi M.G., Yoo H.J., Lee S.J. (2013). Apigenin Induces C-Myc-Mediated Apoptosis in FRO Anaplastic Thyroid Carcinoma Cells. Mol. Cell Endocrinol..

[182] Capece D., Verzella D., Flati I., Arboretto P., Cornice J., Franzoso G. (2022). NF-ΚB: Blending Metabolism, Immunity, and Inflammation. Trends Immunol..

[183] Hoesel B., Schmid J.A. (2013). The Complexity of NF-ΚB Signaling in Inflammation and Cancer. Mol. Cancer.

[184] Shukla S., Gupta S. (2004). Suppression of Constitutive and Tumor Necrosis Factor Alpha-Induced Nuclear Factor (NF)-KappaB Activation and Induction of Apoptosis by Apigenin in Human Prostate Carcinoma PC-3 Cells: Correlation with down-Regulation of NF-KappaB-Responsive Genes. Clin. Cancer Res..

[185] Shukla S., Kanwal R., Shankar E., Datt M., Chance M.R., Fu P., MacLennan G.T., Gupta S. (2015). Apigenin Blocks IKKα Activation and Suppresses Prostate Cancer Progression. Oncotarget.

[186] Hamacher R., Saur D., Fritsch R., Reichert M., Schmid R.M., Schneider G. (2007). Casein Kinase II Inhibition Induces Apoptosis in Pancreatic Cancer Cells. Oncol. Rep..

[187] Pandey M., Kaur P., Shukla S., Abbas A., Fu P., Gupta S. (2012). Plant Flavone Apigenin Inhibits HDAC and Remodels Chromatin to Induce Growth Arrest and Apoptosis in Human Prostate Cancer Cells: In Vitro and in Vivo Study. Mol. Carcinog..

[188] Shukla S., Bhaskaran N., Babcook M.A., Fu P., MacLennan G.T., Gupta S. (2014). Apigenin Inhibits Prostate Cancer Progression in TRAMP Mice via Targeting PI3K/Akt/FoxO Pathway. Carcinogenesis.

[189] Ramaiah M.J., Tangutur A.D., Manyam R.R. (2021). Epigenetic Modulation and Understanding of HDAC Inhibitors in Cancer Therapy. Life Sci..

[190] Chakrabarti M., Banik N.L., Ray S.K. (2013). MiR-138 Overexpression Is More Powerful than HTERT Knockdown to Potentiate Apigenin for Apoptosis in Neuroblastoma in Vitro and in Vivo. Exp. Cell Res..

[191] Jayasooriya R.G.P.T., Kang S.H., Kang C.H., Choi Y.H., Moon D.O., Hyun J.W., Chang W.Y., Kim G.Y. (2012). Apigenin Decreases Cell Viability and Telomerase Activity in Human Leukemia Cell Lines. Food Chem. Toxicol..

[192] Singh V., Sharma V., Verma V., Pandey D., Yadav S.K., Maikhuri J.P., Gupta G. (2015). Apigenin Manipulates the Ubiquitin-Proteasome System to Rescue Estrogen Receptor-β from Degradation and Induce Apoptosis in Prostate Cancer Cells. Eur. J. Nutr..

[193] Chen D., Landis-Piwowar K.R., Chen M.S., Dou Q.P. (2007). Inhibition of Proteasome Activity by the Dietary Flavonoid Apigenin Is Associated with Growth Inhibition in Cultured Breast Cancer Cells and Xenografts. Breast Cancer Res..

[194] Qiu J.G., Wang L., Liu W.J., Wang J.F., Zhao E.J., Zhou F.M., Ji X.B., Wang L.H., Xia Z.K., Wang W. (2019). Apigenin Inhibits IL-6 Transcription and Suppresses Esophageal Carcinogenesis. Front. Pharmacol..

[195] Seo Y.J., Kim B.S., Chun S.Y., Park Y.K., Kang K.S., Kwon T.G. (2011). Apoptotic Effects of Genistein, Biochanin-A and Apigenin on LNCaP and PC-3 Cells by P21 through Transcriptional Inhibition of Polo-like Kinase-1. J. Korean Med. Sci..

[196] Xu Y., Xin Y., Diao Y., Lu C., Fu J., Luo L., Yin Z. (2011). Synergistic Effects of Apigenin and Paclitaxel on Apoptosis of Cancer Cells. PLoS ONE.

[197] Yang C., Song J., Hwang S., Choi J., Song G., Lim W. (2021). Apigenin Enhances Apoptosis Induction by 5-Fluorouracil through Regulation of Thymidylate Synthase in Colorectal Cancer Cells. Redox Biol..

[198] Hu X.Y., Liang J.Y., Guo X.J., Liu L., Guo Y.B. (2015). 5-Fluorouracil Combined with Apigenin Enhances Anticancer Activity through Mitochondrial Membrane Potential (ΔΨm)-Mediated Apoptosis in Hepatocellular Carcinoma. Clin. Exp. Pharmacol. Physiol..

[199] Shao H., Jing K., Mahmoud E., Huang H., Fang X., Yu C. (2013). Apigenin Sensitizes Colon Cancer Cells to Antitumor Activity of ABT-263. Mol. Cancer Ther..

[200] Gao A.M., Ke Z.P., Wang J.N., Yang J.Y., Chen S.Y., Chen H. (2013). Apigenin Sensitizes Doxorubicin-Resistant Hepatocellular Carcinoma BEL-7402/ADM Cells to Doxorubicin via Inhibiting PI3K/Akt/Nrf2 Pathway. Carcinogenesis.

[201] Şirin N., Elmas L., Seçme M., Dodurga Y. (2020). Investigation of Possible Effects of Apigenin, Sorafenib and Combined Applications on Apoptosis and Cell Cycle in Hepatocellular Cancer Cells. Gene.

[202] Singh D., Khan M.A., Mishra D., Goel A., Ansari M.A., Akhtar K., Siddique H.R. (2024). Apigenin Enhances Sorafenib Anti-Tumour Efficacy in Hepatocellular Carcinoma. Transl. Oncol..

[203] Karmakar S., Davis K.A., Choudhury S.R., Deeconda A., Banik N.L., Ray S.K. (2009). Bcl-2 Inhibitor and Apigenin Worked Synergistically in Human Malignant Neuroblastoma Cell Lines and Increased Apoptosis with Activation of Extrinsic and Intrinsic Pathways. Biochem. Biophys. Res. Commun..

[204] Liu X., Zhao T., Shi Z., Hu C., Li Q., Sun C. (2023). Synergism Antiproliferative Effects of Apigenin and Naringenin in NSCLC Cells. Molecules.

[205] Smiljkovic M., Stanisavljevic D., Stojkovic D., Petrovic I., Vicentic J.M., Popovic J., Golic Grdadolnik S., Markovic D., Sanković-Babić S., Glamoclija J. (2017). Apigenin-7-O-Glucoside versus Apigenin: Insight into the Modes of Anticandidal and Cytotoxic Actions. EXCLI J..

[206] Bhosale P.B., Abusaliya A., Kim H.H., Ha S.E., Park M.Y., Jeong S.H., Vetrivel P., Heo J.D., Kim J.A., Won C.K. (2022). Apigetrin Promotes TNFα-Induced Apoptosis, Necroptosis, G2/M Phase Cell Cycle Arrest, and ROS Generation through Inhibition of NF-ΚB Pathway in Hep3B Liver Cancer Cells. Cells.

[207] Sun Q., Lu N.N., Feng L. (2018). Apigetrin Inhibits Gastric Cancer Progression through Inducing Apoptosis and Regulating ROS-Modulated STAT3/JAK2 Pathway. Biochem. Biophys. Res. Commun..

[208] Liu M.M., Ma R.H., Ni Z.J., Thakur K., Cespedes-Acuña C.L., Jiang L., Wei Z.J. (2020). Apigenin 7-O-Glucoside Promotes Cell Apoptosis through the PTEN/PI3K/AKT Pathway and Inhibits Cell Migration in Cervical Cancer HeLa Cells. Food Chem. Toxicol..

[209] Najafipour R., Momeni A.M., Mirmazloomi Y., Moghbelinejad S. (2022). Vitexin Induces Apoptosis in MCF-7 Breast Cancer Cells through the Regulation of Specific MiRNAs Expression. Int. J. Mol. Cell Med..

[210] Czemplik M., Mierziak J., Szopa J., Kulma A. (2016). Flavonoid C-Glucosides Derived from Flax Straw Extracts Reduce Human Breast Cancer Cell Growth In Vitro and Induce Apoptosis. Front. Pharmacol..

[211] Bhardwaj M., Cho H.J., Paul S., Jakhar R., Khan I., Lee S.J., Kim B.Y., Krishnan M., Khaket T.P., Lee H.G. (2017). Vitexin Induces Apoptosis by Suppressing Autophagy in Multi-Drug Resistant Colorectal Cancer Cells. Oncotarget.

[212] Zhang G., Li D., Chen H., Zhang J., Jin X. (2018). Vitexin Induces G2/M-phase Arrest and Apoptosis via Akt/MTOR Signaling Pathway in Human Glioblastoma Cells. Mol. Med. Rep..

[213] Liu X., Jiang Q., Liu H., Luo S. (2019). Vitexin Induces Apoptosis through Mitochondrial Pathway and PI3K/Akt/MTOR Signaling in Human Non-Small Cell Lung Cancer A549 Cells. Biol. Res..

[214] An F., Wang S., Tian Q., Zhu D. (2015). Effects of Orientin and Vitexin from Trollius Chinensis on the Growth and Apoptosis of Esophageal Cancer EC-109 Cells. Oncol. Lett..

[215] Lee C.Y., Chien Y.S., Chiu T.H., Huang W.W., Lu C.C., Chiang J.H., Yang J.S. (2012). Apoptosis Triggered by Vitexin in U937 Human Leukemia Cells via a Mitochondrial Signaling Pathway. Oncol. Rep..

[216] Huang J., Zhou Y., Zhong X., Su F., Xu L. (2022). Effects of Vitexin, a Natural Flavonoid Glycoside, on the Proliferation, Invasion, and Apoptosis of Human U251 Glioblastoma Cells. Oxid. Med. Cell Longev..

[217] He J.D., Wang Z., Li S.P., Xu Y.J., Yu Y., Ding Y.J., Yu W.L., Zhang R.X., Zhang H.M., Du H.Y. (2016). Vitexin Suppresses Autophagy to Induce Apoptosis in Hepatocellular Carcinoma via Activation of the JNK Signaling Pathway. Oncotarget.

[218] Zhao S., Guan X., Hou R., Zhang X., Guo F., Zhang Z., Hua C. (2020). Vitexin Attenuates Epithelial Ovarian Cancer Cell Viability and Motility in Vitro and Carcinogenesis in Vivo via P38 and ERK1/2 Pathways Related VEGFA. Ann. Transl. Med..

[219] Wang L., Klionsky D.J., Shen H.M. (2023). The Emerging Mechanisms and Functions of Microautophagy. Nat. Rev. Mol. Cell Biol..

[220] Kitada M., Koya D. (2021). Autophagy in Metabolic Disease and Ageing. Nat. Rev. Endocrinol..

[221] Deleyto-Seldas N., Efeyan A. (2021). The MTOR-Autophagy Axis and the Control of Metabolism. Front. Cell Dev. Biol..

[222] Chavez-Dominguez R., Perez-Medina M., Lopez-Gonzalez J.S., Galicia-Velasco M., Aguilar-Cazares D. (2020). The Double-Edge Sword of Autophagy in Cancer: From Tumor Suppression to Pro-Tumor Activity. Front. Oncol..

[223] Naponelli V., Modernelli A., Bettuzzi S., Rizzi F. (2015). Roles of Autophagy Induced by Natural Compounds in Prostate Cancer. BioMed Res. Int..

[224] Li X., He S., Ma B. (2020). Autophagy and Autophagy-Related Proteins in Cancer. Mol. Cancer.

[225] Chen Z.S., Tian D., Liao X., Zhang Y., Xiao J., Chen W., Liu Q., Chen Y., Li D., Zhu L. (2019). Apigenin Combined With Gefitinib Blocks Autophagy Flux and Induces Apoptotic Cell Death Through Inhibition of HIF-1α, c-Myc, p-EGFR, and Glucose Metabolism in EGFR L858R+T790M-Mutated H1975 Cells. Front. Pharmacol..

[226] Yang J., Pi C., Wang G. (2018). Inhibition of PI3K/Akt/MTOR Pathway by Apigenin Induces Apoptosis and Autophagy in Hepatocellular Carcinoma Cells. Biomed. Pharmacother..

[227] Lin C.M., Chen H.H., Lin C.A., Wu H.C., Sheu J.J.C., Chen H.J. (2017). Apigenin-Induced Lysosomal Degradation of β-Catenin in Wnt/β-Catenin Signaling. Sci. Rep..

[228] Kim T.W., Lee H.G. (2021). Apigenin Induces Autophagy and Cell Death by Targeting EZH2 under Hypoxia Conditions in Gastric Cancer Cells. Int. J. Mol. Sci..

[229] Lu J., Meng Z., Chen Y., Yu L., Gao B., Zheng Y., Guan S. (2020). Apigenin Induced Autophagy and Stimulated Autophagic Lipid Degradation. Food Funct..

[230] Zeng J., Xie H., Zhang Z.L., Li Z.X., Shi L., Wu K.Y., Zhou Y., Tian Z., Zhang Y., Zhou W. (2022). Apigenin Regulates the Migration, Invasion, and Autophagy of Hepatocellular Carcinoma Cells by Downregulating YAP. Neoplasma.

[231] Janda E., Martino C., Riillo C., Parafati M., Lascala A., Mollace V., Boutin J.A. (2021). Apigenin and Luteolin Regulate Autophagy by Targeting NRH-Quinone Oxidoreductase 2 in Liver Cells. Antioxidants.

[232] Gilardini Montani M.S., Cecere N., Granato M., Romeo M.A., Falcinelli L., Ciciarelli U., D’orazi G., Faggioni A., Cirone M. (2019). Mutant P53, Stabilized by Its Interplay with HSP90, Activates a Positive Feed-Back Loop Between NRF2 and P62 That Induces Chemo-Resistance to Apigenin in Pancreatic Cancer Cells. Cancers.

[233] Lascala A., Martino C., Parafati M., Salerno R., Oliverio M., Pellegrino D., Mollace V., Janda E. (2018). Analysis of Proautophagic Activities of Citrus Flavonoids in Liver Cells Reveals the Superiority of a Natural Polyphenol Mixture over Pure Flavones. J. Nutr. Biochem..

[234] Kayacan S., Yilancioglu K., Akdemir A.S., Kaya-Dagistanli F., Melikoglu G., Ozturk M. (2021). Synergistic Effect of Apigenin and Curcumin on Apoptosis, Paraptosis and Autophagy-Related Cell Death in HeLa Cells. Anticancer Res..

[235] Ghazy E., Taghi H.S. (2022). The Autophagy-Inducing Mechanisms of Vitexin, Cinobufacini, and Physalis Alkekengi Hydroalcoholic Extract against Breast Cancer in Vitro and in Vivo. J. Gastrointest. Cancer.

[236] Battaglia A.M., Chirillo R., Aversa I., Sacco A., Costanzo F., Biamonte F. (2020). Ferroptosis and Cancer: Mitochondria Meet the “Iron Maiden” Cell Death. Cells.

[237] Wang H., Liu C., Zhao Y., Gao G. (2020). Mitochondria Regulation in Ferroptosis. Eur. J. Cell Biol..

[238] Stepanić V., Kučerová-Chlupáčová M. (2023). Review and Chemoinformatic Analysis of Ferroptosis Modulators with a Focus on Natural Plant Products. Molecules.

[239] Feng H., Schorpp K., Jin J., Yozwiak C.E., Hoffstrom B.G., Decker A.M., Rajbhandari P., Stokes M.E., Bender H.G., Csuka J.M. (2020). Transferrin Receptor Is a Specific Ferroptosis Marker. Cell Rep..

[240] Li D., Li Y. (2020). The Interaction between Ferroptosis and Lipid Metabolism in Cancer. Signal Transduct. Target. Ther..

[241] Mou Y., Wang J., Wu J., He D., Zhang C., Duan C., Li B. (2019). Ferroptosis, a New Form of Cell Death: Opportunities and Challenges in Cancer. J. Hematol. Oncol..

[242] Adham A.N., Hegazy M.E.F., Naqishbandi A.M., Efferth T. (2020). Induction of Apoptosis, Autophagy and Ferroptosis by Thymus Vulgaris and Arctium Lappa Extract in Leukemia and Multiple Myeloma Cell Lines. Molecules.

[243] Liu R., Rong G., Liu Y., Huang W., He D., Lu R. (2021). Delivery of Apigenin-Loaded Magnetic Fe_2_O_3_/Fe_3_O_4_@mSiO_2_ Nanocomposites to A549 Cells and Their Antitumor Mechanism. Mater. Sci. Eng. C Mater. Biol. Appl..

[244] Ketelut-Carneiro N., Fitzgerald K.A. (2022). Apoptosis, Pyroptosis, and Necroptosis-Oh My! The Many Ways a Cell Can Die. J. Mol. Biol..

[245] Ai Y., Meng Y., Yan B., Zhou Q., Wang X. (2024). The Biochemical Pathways of Apoptotic, Necroptotic, Pyroptotic, and Ferroptotic Cell Death. Mol. Cell.

[246] Gong Y., Fan Z., Luo G., Yang C., Huang Q., Fan K., Cheng H., Jin K., Ni Q., Yu X. (2019). The Role of Necroptosis in Cancer Biology and Therapy. Mol. Cancer.

[247] Yan J., Wan P., Choksi S., Liu Z.G. (2022). Necroptosis and Tumor Progression. Trends Cancer.

[248] Lee Y.J., Park K.S., Nam H.S., Cho M.K., Lee S.H. (2020). Apigenin Causes Necroptosis by Inducing ROS Accumulation, Mitochondrial Dysfunction, and ATP Depletion in Malignant Mesothelioma Cells. Korean J. Physiol. Pharmacol..

[249] Warkad M.S., Kim C.H., Kang B.G., Park S.H., Jung J.S., Feng J.H., Inci G., Kim S.C., Suh H.W., Lim S.S. (2021). Metformin-Induced ROS Upregulation as Amplified by Apigenin Causes Profound Anticancer Activity While Sparing Normal Cells. Sci. Rep..

[250] Hu G., Li J., Zeng Y., Liu L., Yu Z., Qi X., Liu K., Yao H. (2023). The Anoikis-Related Gene Signature Predicts Survival Accurately in Colon Adenocarcinoma. Sci. Rep..

[251] Taddei M.L., Giannoni E., Fiaschi T., Chiarugi P. (2012). Anoikis: An Emerging Hallmark in Health and Diseases. J. Pathol..

[252] Sattari Fard F., Jalilzadeh N., Mehdizadeh A., Sajjadian F., Velaei K. (2023). Understanding and Targeting Anoikis in Metastasis for Cancer Therapies. Cell Biol. Int..

[253] Adeshakin F.O., Adeshakin A.O., Afolabi L.O., Yan D., Zhang G., Wan X. (2021). Mechanisms for Modulating Anoikis Resistance in Cancer and the Relevance of Metabolic Reprogramming. Front. Oncol..

[254] Hasnat M.A., Pervin M., Lim J.H., Lim B.O. (2015). Apigenin Attenuates Melanoma Cell Migration by Inducing Anoikis through Integrin and Focal Adhesion Kinase Inhibition. Molecules.

[255] Hu X.M., Meng D., Fang J. (2008). Apigenin Inhibited Migration and Invasion of Human Ovarian Cancer A2780 Cells through Focal Adhesion Kinase. Carcinogenesis.

[256] Lee W.J., Chen W.K., Wang C.J., Lin W.L., Tseng T.H. (2008). Apigenin Inhibits HGF-Promoted Invasive Growth and Metastasis Involving Blocking PI3K/Akt Pathway and Beta 4 Integrin Function in MDA-MB-231 Breast Cancer Cells. Toxicol. Appl. Pharmacol..

[257] Zhao M., Ma J., Zhu H.Y., Zhang X.H., Du Z.Y., Xu Y.J., Yu X.D. (2011). Apigenin Inhibits Proliferation and Induces Apoptosis in Human Multiple Myeloma Cells through Targeting the Trinity of CK2, Cdc37 and Hsp90. Mol. Cancer.

[258] Tavsan Z., Kayali H.A. (2019). Flavonoids Showed Anticancer Effects on the Ovarian Cancer Cells: In-volvement of Reactive Oxygen Species, Apoptosis, Cell Cycle and Invasion. Biomed. Pharmacother..

[259] Abid R., Ghazanfar S., Farid A., Sulaman S.M., Idrees M., Amen R.A., Muzammal M., Shahzad M.K., Mohamed M.O., Khaled A.A. (2022). Pharmacological Properties of 4′,5,7-Trihydroxyflavone (Apigenin) and Its Impact on Cell Signaling Pathways. Molecules.

[260] Zhou Y., Yu Y., Lv H., Zhang H., Liang T., Zhou G., Huang L., Tian Y., Liang W. (2022). Apigenin in Cancer Therapy: From Mechanism of Action to Nano-Therapeutic Agent. Food Chem. Toxicol..

[261] Gates M.A., Tworoger S.S., Hecht J.L., De Vivo I., Rosner B., Hankinson S.E. (2007). A Prospective Study of Dietary Flavonoid Intake and Incidence of Epithelial Ovarian Cancer. Int. J. Cancer.

[262] Wang L., Lee I.M., Zhang S.M., Blumberg J.B., Buring J.E., Sesso H.D. (2009). Dietary Intake of Selected Flavonols, Flavones, and Flavonoid-Rich Foods and Risk of Cancer in Middle-Aged and Older Women. Am. J. Clin. Nutr..

